# Effects of spatial constraints of inhibitory connectivity on the dynamical development of criticality in spiking networks

**DOI:** 10.1371/journal.pcbi.1014798

**Published:** 2026-10-01

**Authors:** Felix Benjamin Kern, Takahisa Date, Zenas C. Chao

**Affiliations:** 1 International Research Center for Neurointelligence (WPI-IRCN), UTIAS, The University of Tokyo, Tokyo, Japan; 2 Department of Physics, Graduate School of Science, The University of Tokyo, Tokyo, Japan; University of Nottingham, UNITED KINGDOM OF GREAT BRITAIN AND NORTHERN IRELAND

## Abstract

Neural systems are hypothesized to operate near criticality, enhancing their capacity for optimal information processing, transmission and storage capabilities. Criticality has typically been studied in spiking neural networks and related systems organized in random or full connectivity, with the balance of excitation and inhibition being a key determinant of the critical point of the system. However, given that neurons in the brain are spatially distributed, with their distances significantly influencing connectivity and signal timing, it is unclear how the spatial organization of excitatory and inhibitory connectivity influences the network’s development towards criticality. Here, we systematically constrain the distance and density of inhibitory connectivity in two-dimensional spiking networks and allow synaptic weights to develop with activity-dependent excitatory and inhibitory plasticity in the presence of a low level of stochastic intrinsic activity. We then investigate the relationship between inhibitory connectivity, synaptic weights, and the resulting network activity during and after development. We find that networks with longer-range inhibitory synapses tend towards more supercritical behavior compared to networks with a similar number of shorter-range inhibitory synapses. We show that this distance dependence is a consequence of weaker long-range synapses after development due to the presence of synaptic delays, which shift most spike pairs outside of the potentiation window of the inhibitory learning rule.

## Author summary

The brain is thought to operate near a critical state, a regime that supports efficient information processing, transmission, and storage. While previous studies have shown that the balance between excitation and inhibition is important for maintaining criticality, much less is known about how the spatial organization of neural circuits contributes to this process. Using a spatially embedded spiking neural network with biologically motivated synaptic plasticity, we investigated how the range of inhibitory connections influences the development of critical dynamics. We found that networks with predominantly long-range inhibitory connections consistently developed more supercritical activity than networks with similar numbers of short-range inhibitory connections. This effect arose because axonal conduction delays shifted spike timing outside the potentiation window of inhibitory spike-timing-dependent plasticity, preventing distant inhibitory synapses from strengthening effectively. As a result, long-range inhibition provided weaker functional control of network activity despite identical structural connectivity. Our findings identify transmission delays as an important developmental factor linking the spatial organization of inhibitory circuits to network criticality. They further suggest that developmental alterations in inhibitory connectivity may influence brain dynamics by changing the effectiveness of inhibitory interactions, providing experimentally testable predictions for studies of cortical development and neurodevelopmental disorders.

## Introduction

The brain criticality hypothesis states that neurons in the brain are organized in such a way that their collective activity lies at or near the phase transition between ordered and random activity [1,2]. At this critical point, neural activity is characterized by self-similarity and scale-free activity patterns. Criticality in neural networks has been shown to be beneficial in many ways. Criticality leads to optimal computational capacities [3,4] and optimal information transmission [5,6]. Networks operating at or near criticality exhibit high information storage capacity [6–8] and achieve their maximum dynamic range, making them highly sensitive to changes in external stimulation [9,10].

Criticality was first discussed in the field of physics with the sandpile model. There, avalanche event sizes are scale-free, meaning that there is a power-law relationship between the size of avalanches and their frequency or probability of occurrence. Scale-free neural activity, so-called neuronal avalanches, were subsequently discovered in cortical neurons as a signature of criticality [5]. Neuronal avalanches have been observed in various in-vitro and in-vivo experimental models [5,11,12].

How the brain develops into the critical state is an active research question [12–14]. Previous research has revealed that network structure, long-term synaptic plasticity rules, and homeostatic mechanisms can drive networks to develop into a critical state [15–18]. Meanwhile, short-term plasticity is known to expand the critical regime from a finely balanced point to a broad region in parameter space [15,19]. Finally, regardless of how criticality is established, the single most important parameter for its maintenance is the balance of excitation and inhibition (E/I balance) within a network [20–22].

Most research into criticality has been conducted in randomly or fully connected networks, ignoring the spatial dimensionality of the brain and the resulting structural anisotropies. The effect of spatial constraints has been investigated in the context of excitatory connectivity [20,23], while synaptic delays have been shown to affect how quickly networks settle into a steady state [24]. However, to our knowledge, there has been no work investigating the impact of inhibitory connectivity or of the influence of conduction delays on the steady-state activity itself.

Here, we investigate the effect of spatial constraints in inhibitory connectivity on the long-term development of criticality, marking a first foray into a more comprehensive treatment of criticality in networks with spatial dimensions. In addition, unlike related studies using spatial constraints for connectivity only [20,23,25], we explicitly model the delays imposed by conduction along axons and find that such delays have a profound effect on the development of synaptic weights.

## Methods

### Neuron model

We chose a basic leaky integrate-and-fire neuron model with a membrane potential u evolving according to


$$\begin{array}{c} \tau \frac{du}{dt}=\left(u_{r}-u\right)+R_{m}I_{syn}(t)+\sigma \sqrt{\text{2}\tau }\xi (t) \end{array}$$

with membrane time constant τ=30 ms, membrane resistance $R_{m}=\text{1}\text{0}\text{0}$ MΩ, and resting membrane potential $u_{r}=-\text{6}\text{9}$ mV. When a neuron’s membrane potential reached the firing threshold θ=−54 mV, a spike was emitted, and the membrane potential was clamped to $u=u_{\text{0}}=-\text{7}\text{4}$ mV for 3 ms (excitatory neurons) or 2 ms (inhibitory neurons). Noise was injected into the membrane potential in the form of an Ornstein-Uhlenbeck process with a Gaussian random variable ξ(t) and a standard deviation σ=5 mV, yielding an intrinsic firing rate of approximately $\text{0}.\text{2}\text{8}{\text{s}}^{-\text{1}}$. A sample membrane potential trace of an isolated excitatory neuron is shown in Fig 1A.

**Fig 1:**
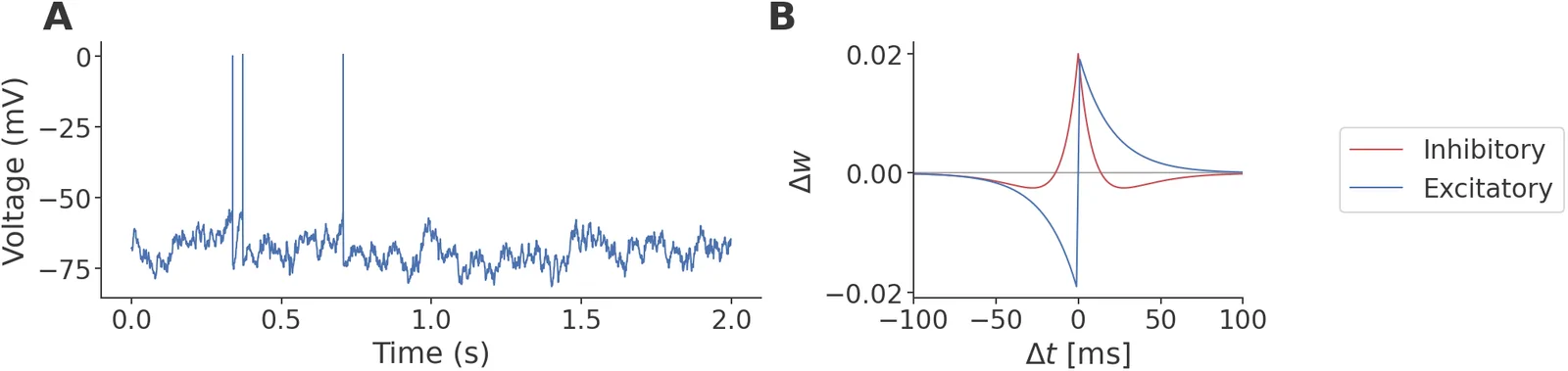
Model dynamics. **(A)** Sample neuronal voltage trace of a disconnected excitatory neuron. **(B)** Excitatory and inhibitory STDP rules, with $\Delta t=t_{post}-t_{pre}$.

### Synapse model

Synaptic currents were modeled in a conductance-based manner, following


$$\begin{array}{c} I_{syn}=g_{exc}\left(E_{exc}-u\right)+g_{inh}\left(E_{inh}-u\right) \end{array}$$

with excitatory reversal potential $E_{exc}=\text{0}$ mV and inhibitory reversal potential $E_{inh}=-\text{1}\text{0}\text{0}$ mV. Synaptic conductances evolved according to


$$\begin{array}{c} \tau_{exc}\frac{dg_{exc}}{dt}=-g_{exc}+U\overset{―}{g}\sum_{j\in E}x_{j}w_{ij}\delta \left(t-{\hat{t}}_{j}\right) \end{array}$$

and


$$\begin{array}{c} \tau_{inh}\frac{dg_{inh}}{dt}=-g_{inh}+U\overset{―}{g}\sum_{j\in I}Ux_{j}w_{ij}\delta (t-{\hat{t}}_{j}) \end{array}$$

for excitatory and inhibitory synapses, respectively, with excitatory time constant $\tau_{exc}=\text{2}$ ms, inhibitory time constant $\tau_{inh}=\text{4}$ ms, scale factor $\overset{―}{g}=\text{8}\text{0}$ nS, the sets of presynaptic excitatory and inhibitory neurons E and I, respectively, synaptic weight $w_{ij}$, the Dirac delta function δ(·) and presynaptic spike times ${\hat{t}}_{j}$.

Synapses were subject to short-term depression (STD), using a deterministic simplification of the model in [26]. We modeled the depression variable $x_{j}$ as a property of the presynaptic neuron j,


$$\begin{array}{c} \tau_{x}\frac{dx_{j}}{dt}=\left(\text{1}-x_{j}\right)-Ux_{j}\delta (t-{\hat{t}}_{j}) \end{array}$$

with recovery time constant $\tau_{x}=\text{1}\text{5}\text{0}$ ms and release fraction U=0.4.

Finally, all synapses were also governed by long-term spike-timing dependent plasticity (STDP). In excitatory synapses, we used standard STDP [27], with updates to the dimensionless weight value $w_{ij}\in [\text{0},\text{1}]$ of the excitatory synapse from neuron j to neuron i following


$$\begin{array}{c} \Delta w=\sum_{t_{j}}\sum_{t_{i}}F\left(t_{i}-t_{j}\right) \end{array}$$

where


$$\begin{array}{c} F(\Delta t)=\left\{ \begin{array}{cc} A_{p}e^{\frac{\Delta t}{\tau_{p}}},\; & \Delta t>\text{0}\\ {-A}_{p}e^{-\frac{\Delta t}{\tau_{p}}},\; & \Delta t\leq \text{0} \end{array}\right. \end{array}$$

with $\tau_{p}=\text{2}\text{0}$ ms and $A_{p}=\text{0}.\text{0}\text{1}$. To avoid any doubt, we use $\Delta t=t_{post}-t_{pre}$.

For inhibitory synapses, we employed a symmetrical Hebbian STDP rule [16,28] whose weight update function follows


$$\begin{array}{c} F(\Delta t)=A_{f}e^{\frac{-|\Delta t|}{\tau_{f}}}-A_{s}e^{\frac{-|\Delta t|}{\tau_{s}}} \end{array}$$

where $A_{f}=\text{0}.\text{0}\text{2}$, $A_{s}=\text{0}.\text{0}\text{1}$, and the time constants $\tau_{f}=\text{1}\text{0}$ ms and $\tau_{s}=\text{2}\text{0}$ ms. This rule gives rise to a “Mexican hat” shaped weight update function resembling the homeostatic rule in Vogels et al. [29]. A similar symmetric iSTDP rule has been observed experimentally in the mouse auditory cortex [30]. Both excitatory and inhibitory rules are illustrated in Fig 1B. Weight updates were calculated online whenever a pre- or postsynaptic spike arrived at the synapse (see below), using exponentially decaying activity traces as demonstrated in Song, Miller, and Abbott [27]. A hard bound of $w_{ij}\in [\text{0},\text{1}]$ was enforced by setting any negative $w_{ij}$ to 0 and setting any $w_{ij}>\text{1}$ to 1 immediately after the weight update.

Spikes were delivered to synapses with a distance-dependent axonal delay corresponding to a conduction velocity of 0.15 m/s along a direct linear path from presynaptic to postsynaptic neuron location.

### Network structure

We investigated networks of $n_{exc}=\text{8}\text{0}$ excitatory and $n_{inh}=\text{2}\text{0}$ inhibitory neurons scattered randomly across a two-dimensional disk with a radius of 2 mm. Network structure was established based on the spatial proximity of neurons, as illustrated in Fig 2A. Specifically, for a given presynaptic neuron of type k∈{exc,inh}, all neurons located within a radius of $r_{k}$ were considered potential postsynaptic targets. From among this subset, synaptic connections were established at random with a uniform probability $p_{k}$. While excitatory connectivity was fixed at $r_{exc}=\text{1}.\text{3}$ mm and $p_{exc}=\text{0}.\text{6}$, we varied inhibitory connectivity systematically, with $r_{inh}\in \{\text{0}.\text{5},\text{1},\text{2},\text{3},\text{4}\}$ mm and $p_{inh}\in \{\text{0}.\text{1},\text{0}.\text{3},\text{0}.\text{5},\text{0}.\text{7},\text{1}\}$, yielding a 5-by-5 grid of parameter settings to explore.

**Fig 2:**
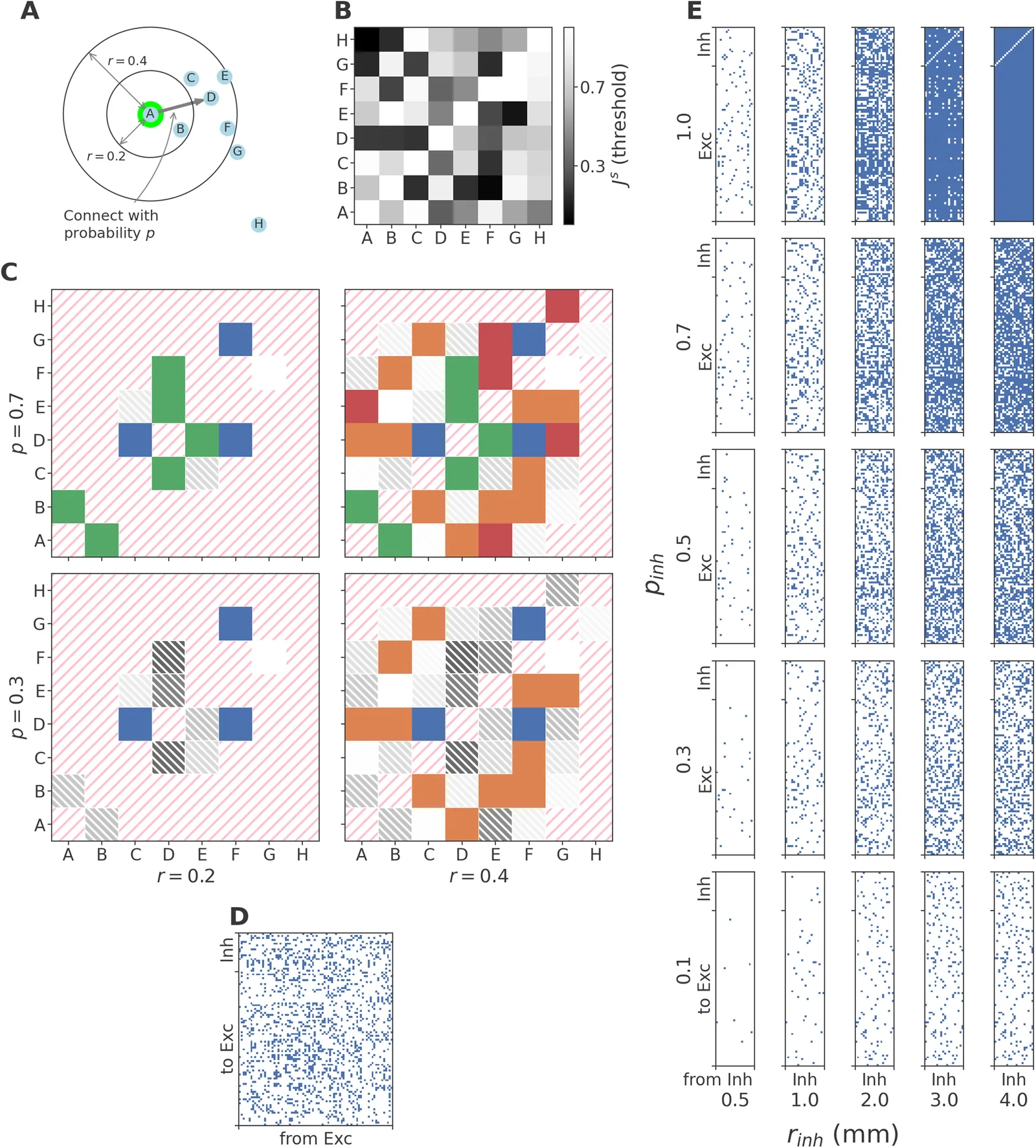
Illustration of the additive process used to generate connectivity. **(A)** Locations of the eight neurons (A-H) used in this illustration. Black circles are drawn at radius r=0.2 and r=0.4 around the highlighted neuron A for reference. **(B)** The adjacency matrix scaffold $J^{s}$ for this illustration, drawn pseudorandomly from a uniform distribution. Darker squares will be connected at lower probability thresholds p. **(C)** The resulting adjacency matrices for two values each of r and p. Neuron pairs that are too distant (i.e., their distance is greater than r) are disconnected, along with the diagonal (i.e., no autapses), as illustrated by the red hatching. Neuron pairs that are within the spatial window for r, but whose value of $J^{s}$ exceeds p, are disconnected and shown hatched in grey (same color scale as in panel **B)**. Neuron pairs that meet the criteria for both distance (<r) and probability ($J^{s}<p$) are connected and shown in solid color, with different hues indicating connections that are present throughout (blue), added by increasing r (green), increasing p (orange), or increasing both r and p together (red). **(D-E)** Adjacency matrices of one network “prototype” (see text). Blue pixels indicate existing connections. **(D)** Excitatory connectivity, drawn from $r_{exc}=\text{1}.\text{3}$ mm and $p_{exc}=\text{0}.\text{6}$. **(E)** Inhibitory connectivity across the 5x5 grid of values for $r_{inh}$ and $p_{inh}$.

To minimize the impact of structural stochasticity, we opted to maintain as much of the structure as possible across parameter settings. Specifically, spatial positions and excitatory connections were maintained across parameter settings, while the inhibitory connections were constructed in an additive fashion with increasing $r_{inh}$ and $p_{inh}$. To achieve this, we initialized a scaffold $J_{inh}^{s}\in [\text{0},\text{1}{]}^{(n_{exc}+n_{inh})\times n_{inh}}$ of the adjacency matrix $J_{inh}$ with a single set of pseudo-random numbers for all 25 parameter settings. Then, we selected connections using the specific values of $r_{inh}$ and $p_{inh}$, defining the elements of $J_{inh}$ as


$$\begin{array}{c} J_{ji}=\left\{ \begin{array}{cc} \text{1}, & \text{if }dist(i,j)<r_{inh}\text{ and }J_{ji}^{s}<p_{inh}\\ \text{0}, & \text{otherwise} \end{array}\right. \end{array}$$

In this way, the set of inhibitory connections in each network is a subset of the connections in all networks with greater or equal $r_{inh}$ and $p_{inh}$. The principle of this approach is illustrated with a toy example with eight neurons in Fig 2. Fig 2A shows the spatial locations of the neurons, Fig 2B a randomly drawn adjacency matrix scaffold $J^{s}$, and Fig 2C shows the resulting adjacency matrices for two values of r and p each.

We use the term “network prototype” to refer to a single set of 25 networks created in this way, each sharing in common the neuron locations, excitatory connectivity, and parts of inhibitory connectivity. We independently generated and simulated a total of 10 network prototypes for analysis. The adjacency matrices of one of these are presented in Fig 2D, showing the excitatory portion, which is identical across parameter settings, and in Fig 2E, showing the inhibitory portions across the 5x5 grid of $r_{inh}$ and $p_{inh}$.

### Development

While the structure of the networks, i.e., the presence or absence of synapses, was fixed, we initialized all synaptic weights to zero to imitate an immature neuronal network or silent synapses [31]. We then simulated each network for 15 hours, allowing weights to develop according to the plasticity rules outlined above. To account for the influence of stochasticity in intrinsic neuronal activity, each simulation was repeated five times with different random seeds.

### Criticality

To investigate criticality properties of the developing networks, we analyzed the neural avalanches and calculated the criticality variable Δp as follows. First, we counted spikes across the network in bins of 1 ms duration. Contiguous periods of non-zero spike counts were defined as “avalanches,” with size S corresponding to the total number of spikes and duration T corresponding to the total length of contiguous bins. Within temporal windows of 5 minutes, we calculated the probability p(s) of observing an avalanche of a given size and approximated the resulting distribution with a power-law function, $p(S)=aS^{-\alpha }$, using linear least-squares fitting in log–log space (i.e., fitting between log(S) and log(p(S))). Because the avalanche size distribution is approximately linear in the small-size region, we restricted the fitting range to $S\leq S_{max}=\text{1}\text{0}$. This approach was adapted from [32], in which both the small- and large-size tails were excluded from fitting because of their nonlinearity. In our case, only the large-size tail was excluded, as the small-size region remained approximately linear. To assess the robustness of this choice, we also performed sensitivity analyses using $S_{max}=\text{5},\text{2}\text{0}$, confirming that the conclusions obtained with $S_{max}=\text{1}\text{0}$were not dependent on the specific fitting range selected.

Finally, the desired measure Δp was defined as the average deviation of the data from this fit across all avalanche sizes, i.e.,


$$\begin{array}{c} \Delta p=\left\langle \text{log}\left(p(S)\right)-\text{log}(aS^{-\alpha })\right\rangle \end{array}$$

In addition to Δp, we calculated κ, the deviation from a theoretical avalanche size distribution with an exponent b of -1.5, which corresponds to the scaling relation found in living networks [10,20].

As an alternative measure of criticality, we examined the relationships between scaling exponents. In addition to fitting the avalanche size distribution $p(S)\sim S^{-\alpha }$, we fitted the lifetime distribution $p(T)\sim T^{-\tau }$ by performing a linear regression on log(T) versus log(p(T)) for $T\leq T_{max}=\text{1}\text{0}$. We also analyzed the avalanche size conditioned on lifetime, $S(T)\sim T^{\text{1}/\sigma \nu z}$, by linearly fitting log(S(T)) against log(T). Using these fitted exponents, we computed the scaling relation


$$\begin{array}{c} \gamma =\left|\frac{\tau -\text{1}}{\alpha -\text{1}}-\frac{\text{1}}{\sigma \nu z}\right| \end{array}$$

In systems at criticality, this relation is expected to hold exactly, such that γ=0 [33,34].

We also evaluated the Fano factor$F(T)=\frac{\left\langle N^{\text{2}}(t,t+T)\right\rangle -{\left\langle N(t,t+T)\right\rangle }^{\text{2}}}{\left\langle N(t,t+T)\right\rangle }$where N(t,t+T) corresponds to the number of spikes fired across the network in the time between t and t+T. In critical processes, F(T) follows a power law [12].

## Results

Our results are presented as follows. First, we will briefly cover the development of the networks over 15 hours of simulation time, giving an intuition for the steady-state activity patterns and their emergence. Then, we will focus on the steady state, comparing activity patterns across different parameter settings and confirming that our outcomes broadly agree with previous findings on the boundary conditions for criticality. Finally, we will investigate how the spatial constraint on inhibitory connections affects criticality, showing that networks with more long-range inhibitory connections tend to be more supercritical than networks with more short-range connections, since long-range inhibitory synapses are weaker than short-range synapses and therefore less able to counter excitatory influence.

### Development

Starting from an initial state with connections established but ineffective (w = 0), networks followed a largely conserved trajectory of changes in weights and activity irrespective of the constraints on inhibitory connectivity. Fig 3 shows an example, elaborated below, of this trajectory in three networks. Other runs (i.e., simulations of the same network with different random seeds for intrinsic activity) and other networks and network prototypes followed a qualitatively very similar profile.

**Fig 3:**
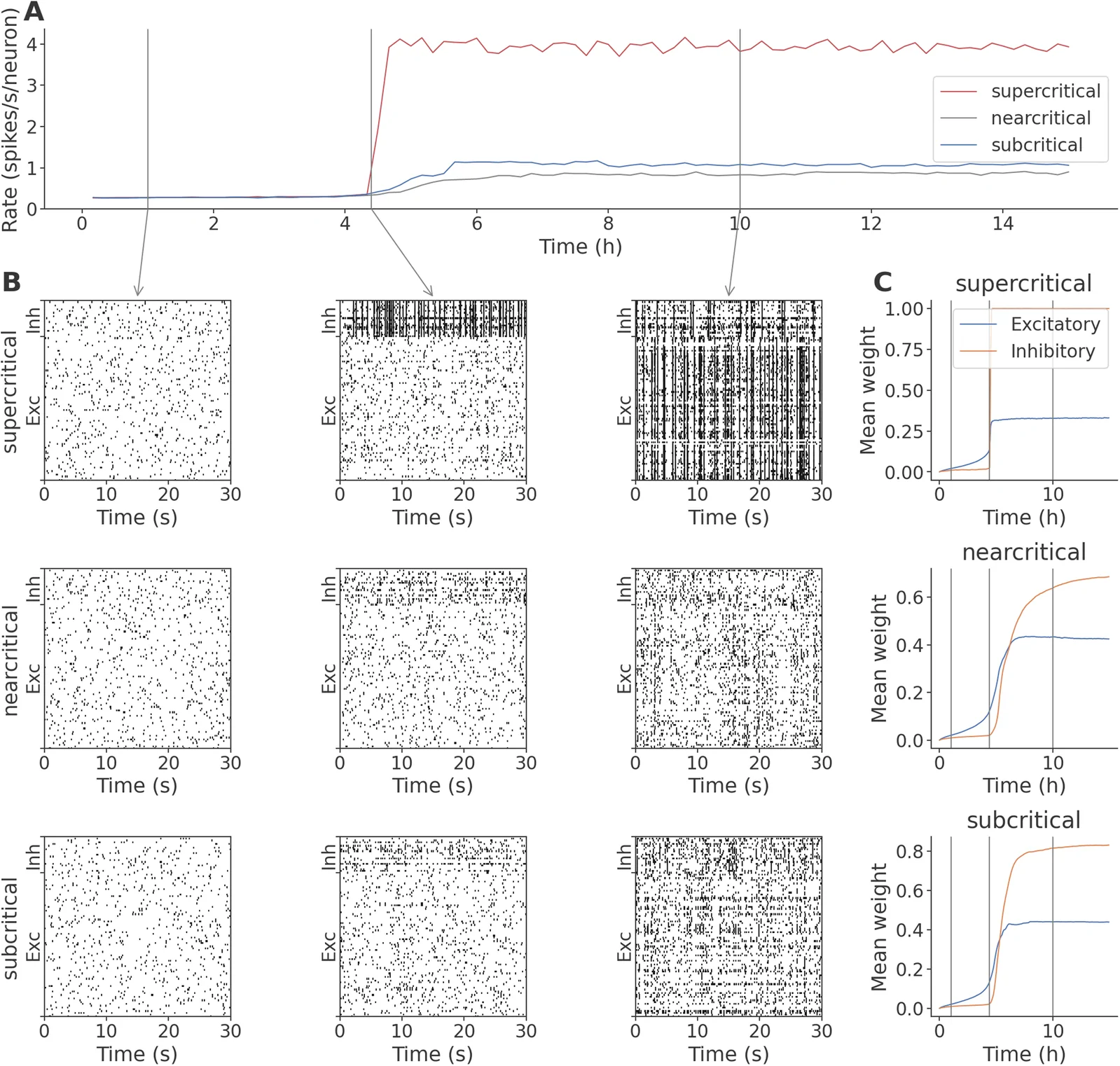
Development of three sample networks (supercritical: $r_{inh}=0.5$ mm, $p_{inh}=0.3$, nearcritical: $r_{inh}=4.0$ mm, $p_{inh}=1.0$, subcritical: $r_{inh}=2.0$ mm, $p_{inh}=1.0$). **(A)** Mean firing rate in spikes per second per neuron. Red: supercritical. Gray: near-critical. Blue: subcritical. **(B)** Raster plots of network activity during 30 second samples taken from the time points indicated in panel A (1h, 4h20min, 10h). **(C)** Mean synaptic weights across existing excitatory connections (blue) and inhibitory connections (orange).

Initial activity was sparse, uncoordinated, and driven exclusively by intrinsic voltage fluctuations (Fig 3A and 3B). After four to five hours, excitatory weights (Fig 3C) had grown sufficiently strong to cause coordinated activity, which quickly escalated to highly synchronized network-wide bursting (Fig 3B, middle row). This, in turn, drove inhibitory weights to catch up, providing a degree of counterbalance and allowing networks to control activity into an approximately steady state within one to two hours (Fig 3B, right row). Both firing rate and weights then remained approximately constant until the end of the 15-hour simulation period.

### Criticality after development

To assess the activity patterns at steady state, we focused on the final two hours of simulation (13–15 h) in each network and run. Fig 4A shows raster plots of one network prototype, taken from the final 20 seconds in each of the 25 settings of $r_{inh}$ and $p_{inh}$. In Fig 4B, we illustrate the derivation of the criticality measure Δp on data from three of these networks, exhibiting supercritical behavior (left, $r_{inh}=\text{0}.\text{5}$ mm and $p_{inh}=\text{0}.\text{1}$, notice the large number of large avalanches), approximately critical behavior (center, $r_{inh}=\text{2}$ mm and $p_{inh}=\text{0}.\text{5}$), and subcritical behavior (right, $r_{inh}=\text{4}$ mm and $p_{inh}=\text{1}$, with few large avalanches).

**Fig 4:**
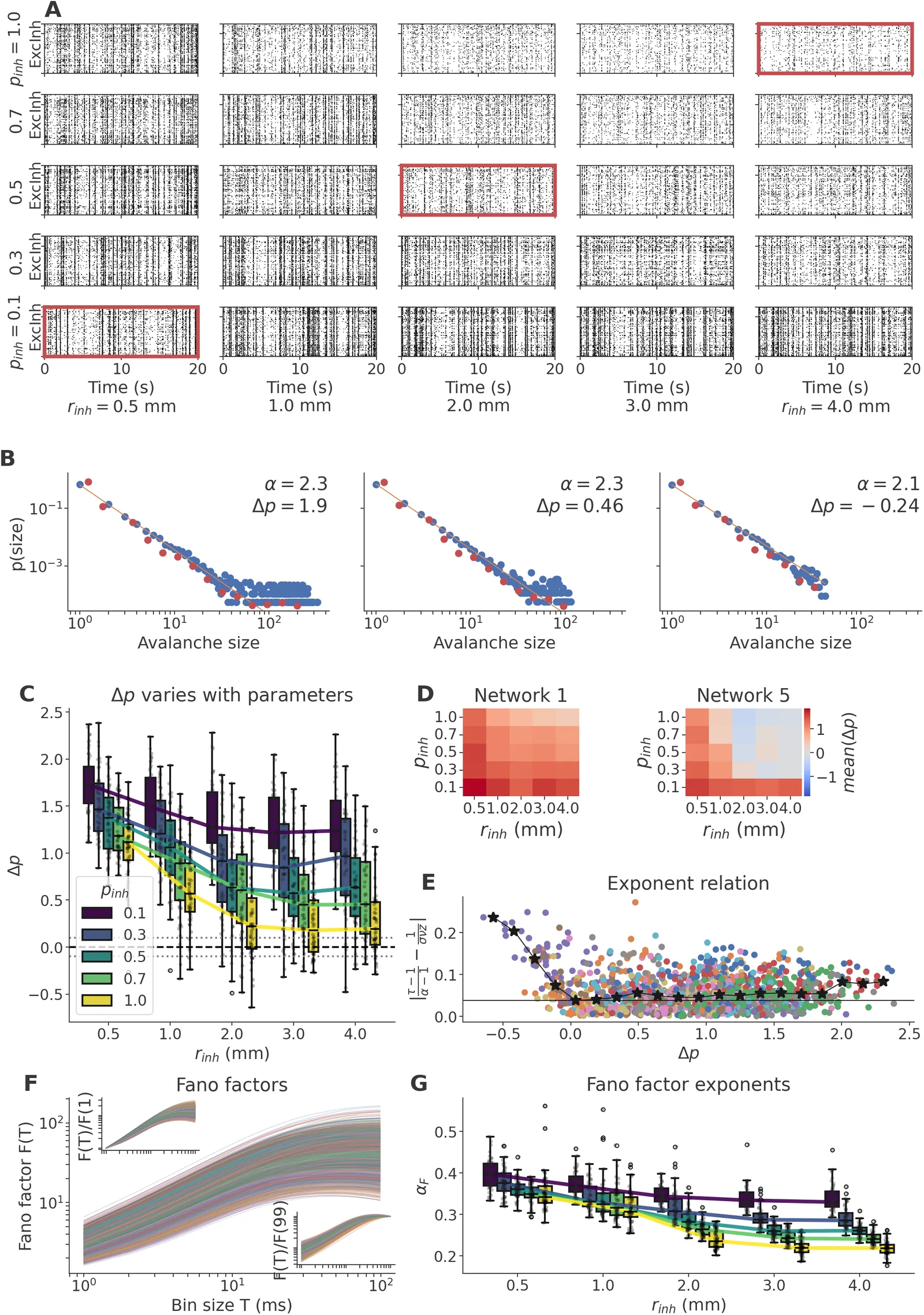
Criticality after development. **(A)** Spike raster plots showing the firing patterns of a sample network prototype across the grid of $r_{inh}$ and $p_{inh}$ after 15 hours of development. **(B)** Frequency of avalanches plotted against their respective size (blue points: evenly spaced binning, red points: 20 log binning) along with the corresponding power-law fit (solid line), illustrating the calculation of Δp, which is the mean error between the observed frequencies and the power-law fit. The three plots correspond to the highlighted raster plots in panel A and summarize the final 5 minutes of activity in each of these networks. Inset: Exponent *𝛼* and criticality outcome Δp. **(C)** Distribution of Δp across the grid of $r_{inh}$ and $p_{inh}$. Δp was calculated in each run of each network from activity between 13 and 15 hours. Boxes show quartiles, with whiskers extending to points within 1.5 times the interquartile range. Boxes are clustered by the (categorical) value of $r_{inh}$ and color-coded by $p_{inh}$. Each box summarizes 5 runs × 10 networks created from a pair of r_inh and $p_{inh}$. **(D)** The mean of Δp across all runs in each grid cell in two sample networks. **(E)** The criticality relation $\left|\frac{\tau -\text{1}}{\alpha -\text{1}}-\frac{\text{1}}{\sigma \nu z}\right|$ plotted against Δp, with color indicating individual network prototypes. Connected stars: median trace of criticality relation plotted at 20 linearly spaced bin centers with black stars. **(F, G)** Fano factors plot. **F**: 𝐹(𝑇) calculated across a range of 1 to 100 ms. 𝐹(𝑇) plotted against bin size 𝑇, showing power-law scaling up to about 30-40 ms. Each line corresponds to a network instantiation, colors are arbitrary. Inset plots normalizing each curve to the minimum (upper left, 1 ms) and maximum (lower right, 99 ms) interval investigated. **G**: Exponents of the power-law fit $F(T)\sim T^{\alpha_{F}}$ across the parameter space (color-coded by $p_{inh}$in the same style as panel **C)**, showing a clear progression towards lower exponents with increased inhibitory density and radius.

The average values of Δp in the final two hours for each of the 25 parameter configurations are shown individually in Fig 4C. The means of Δp across runs in two sample networks, indicative of the expected outcome for each setting of $r_{inh}$ and $p_{inh}$, are shown in Fig 4D. We observed supercritical behavior throughout most of the parameter space, being the majority outcome in nearly all conditions, except where inhibitory connectivity was very dense with $r_{inh}\geq \text{2}$ mm and $p_{inh}=\text{1}$. Criticality, which we define as |Δp|<0.1, was seen to emerge infrequently in most parameter settings, but it occurred more frequently at high inhibitory connectivity densities. A similar picture emerged from the analysis of κ, which measures the deviation of the avalanche size distribution from a theoretical distribution with exponent -1.5 (see S1 Fig). This measure likewise showed more subcritical outcomes in the upper right quadrant than in other regions of the $r_{inh}$-$p_{inh}$ space. In sum, the higher the density of inhibitory connections at the outset, the lower the value of Δp became after development.

To assess whether our use of Δp is appropriate, we independently calculated the criticality relation $\gamma =\left|\frac{\tau -\text{1}}{\alpha -\text{1}}-\frac{\text{1}}{\sigma \nu z}\right|$, which tends towards 0 for critical networks (see S1 Fig). As shown in Fig 4E, the Δp-γplot shows a minimum around Δp=0, indicating that the two measures largely agree about the extent of criticality in our setup.

Furthermore, we plotted Fano factors in Fig 4F and 4G. Fano factor plots suggests network states close to criticality throughout the parameter space, but only at very short time scales, with levels of activity clustering lower than expected from critical systems at time scales above 30–40 ms.

In the power-law fitting used to calculate Δp, we introduced upper bounds on the fitting range: avalanche size $S\leq S_{\text{max}}=\text{1}\text{0}$and duration $T\leq T_{\text{max}}=\text{1}\text{0}$. To assess the sensitivity of Δp to these fitting ranges, we repeated the fitting with $S_{\text{max}}=T_{\text{max}}=\text{5}$or 20. As shown in S2 Fig, both the Δp distribution and the Δp–γ relationship are qualitatively preserved, indicating that our analysis is robust to the choice of $S_{\text{max}}$ and $T_{\text{max}}$.

In the following, we will analyze how the spatial constraint imposed on our networks leads to this outcome in the developed state. First, we will confirm that the level of criticality is related to synaptic weight balance, as numerous studies have found (Fig 5). Then, we will take a closer look at the relationship between the spatial constraint – and in particular, the length of inhibitory connections – and the criticality level (Fig 6). Finally, in Figs 7 and 8, we will observe how inhibitory connection length relates to weight balance, leading to a detailed explanation of how greater inhibitory connection length leads to higher levels of Δp.

**Fig 5:**
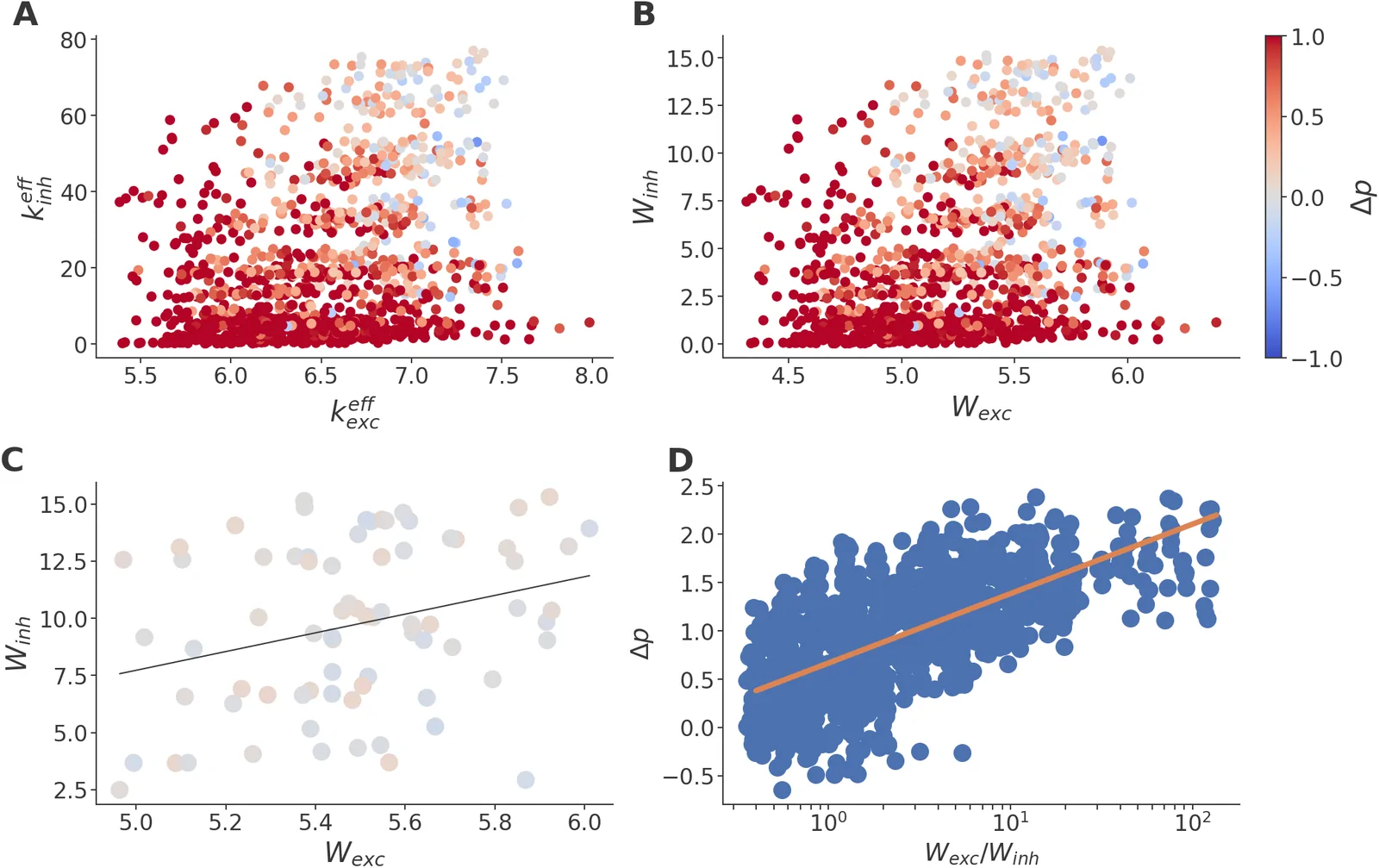
Balance of excitation and inhibition is tightly linked to criticality. **(A)** Network degree, i.e., the mean number of incoming connections with w≥0.5 per neuron, separated by the type of incoming connection and plotted against each other. Each dot represents one network run in its weight configuration after 15 hours of development. Color represents Δp. **(B)** Total weight of excitatory and inhibitory synapses (without applying the weight threshold), averaged across neurons and plotted against each other in the same manner as in panel **A. (C)** The same data in the subset of networks with |Δp|<0.1. The black line is the linear least-squares fit to these data points with slope 4.097 and intercept -12.76. **(D)** The criticality outcome Δp plotted against the ratio of excitatory to inhibitory weight totals, along with a log-linear regression line.

**Fig 6:**
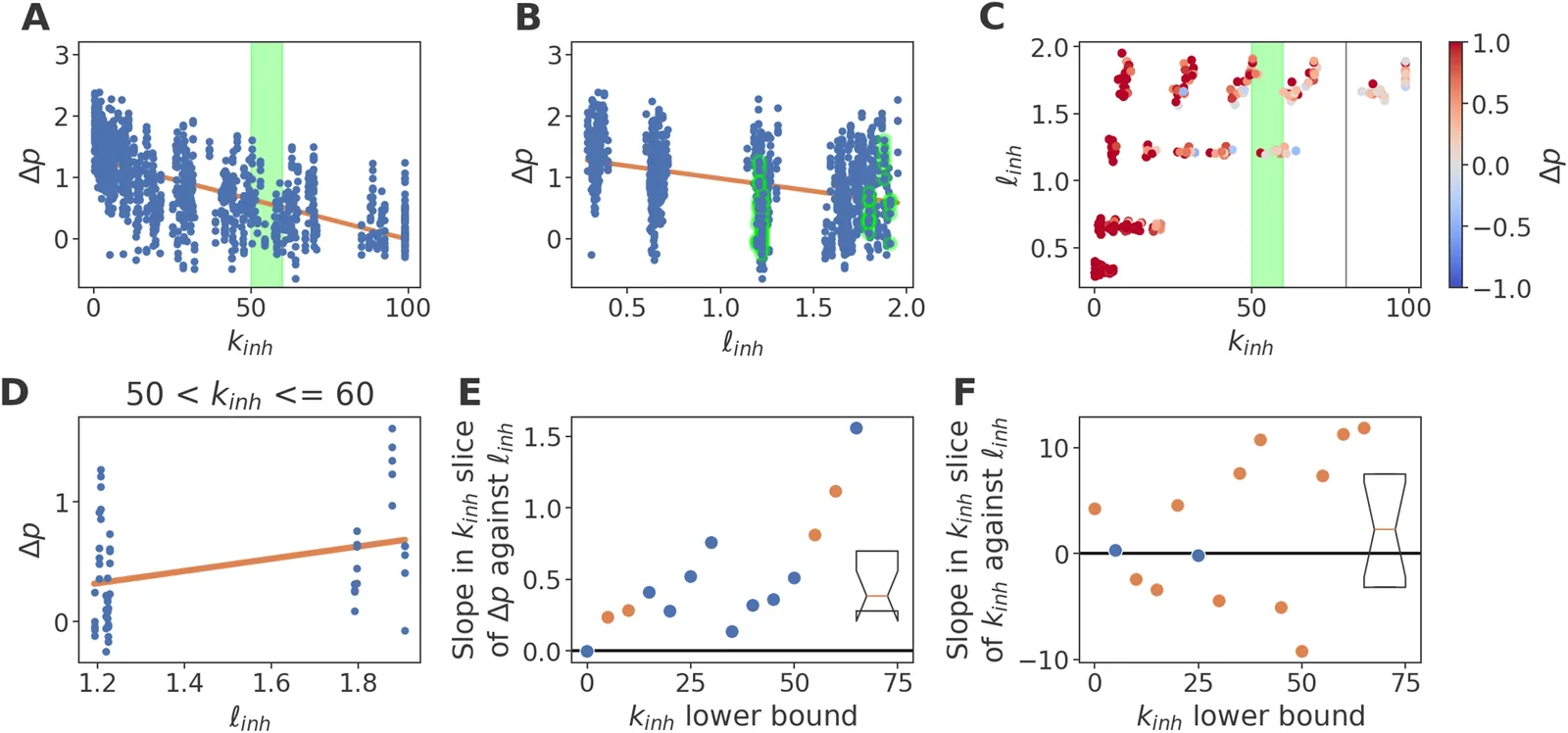
Long-range inhibitory networks yield larger avalanches. **(A)** Criticality outcome (Δp) plotted against the mean inhibitory degree (structurally defined $k_{inh}$, i.e., including all existing connections regardless of weight) along with a linear fit (orange). The green box highlights one slice of $k_{inh}$ containing networks of approximately equal degree referred to in other panels. **(B)** Criticality outcome plotted against mean length of inhibitory connections (structurally defined $l_{inh}$) along with a linear fit. Points highlighted in green correspond to the slice in panel **A. (C)** Criticality outcome (Δp, point color) shown as a function of $k_{inh}$ and $l_{inh}$. The green rectangle highlights the slice in panel A, and the vertical line indicates the maximum $k_{inh}$ used; the few networks with higher $k_{inh}$ were not considered in the subsequent panels. **(D)** Sample correlation between $l_{inh}$ and Δp in the slice indicated in panels A-C. The orange line indicates the best-fit linear model. Correlation coefficient 0.34. 𝑝 = 0.01. **(E)** Slopes of the linear fit relating $l_{inh}$to Δp in each slice of$k_{inh}$. Orange color indicates significant linear correlations (Bonferroni-corrected 𝑝 < 0.05). The box plot at the right shows the interquartile range (box), median (line) and 95% CI of the median (notch) of these slopes. **(F)** Slopes of the linear fit relating $l_{inh}$to $k_{inh}$ in each slice of$k_{inh}$. Orange color indicates significant linear correlations (Bonferroni-corrected 𝑝 < 0.05).

**Fig 7:**
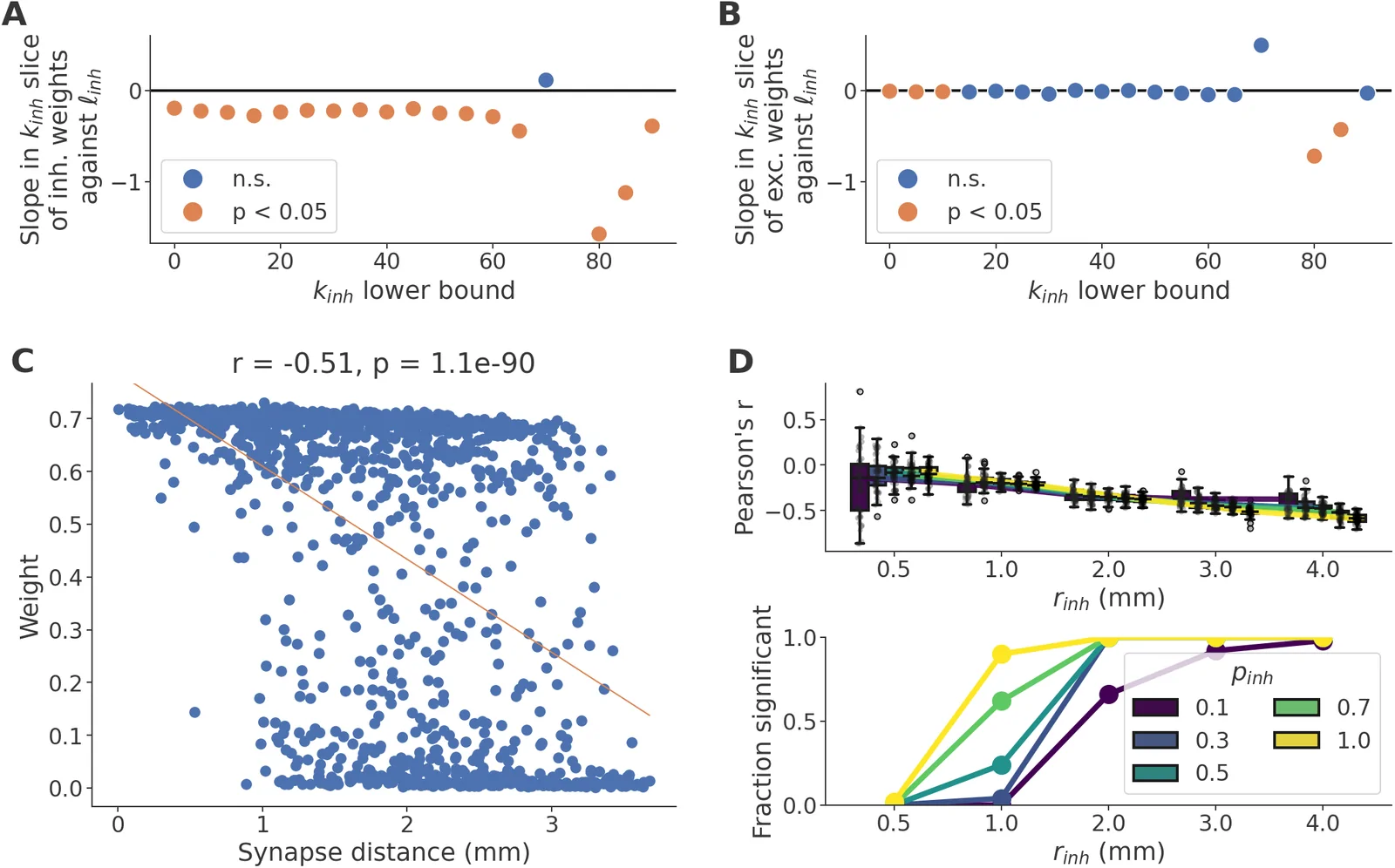
Long-range inhibitory connections are less functional. **(A)** Slopes of linear fits relating $l_{inh}$to average inhibitory weights in each slice of $k_{\text{inh}}$. See Fig 6 for an explanation of the slices and S5 – S8 Figs for scatter plots of each slice. Orange color indicates significant linear correlations (Bonferroni-corrected p < 0.05). **(B)** Slopes of linear fits relating $l_{inh}$to average excitatory weights in each slice of $k_{\text{inh}}$. **(C)** Relationship between inhibitory weight and synaptic distance within networks. The weight of inhibitory synapses (mean across development) is plotted against the distance between the corresponding neurons in a representative sample network ($r_{inh}=\text{4}$ mm, $p_{inh}=\text{0}.\text{7}$; $k_{\text{inh}}=\text{6}\text{8}$). The orange line represents the linear fit. **(D)** Top: Distributions of Pearson’s *r* of this correlation within networks (i.e., between weight and distance of inhibitory synapses). Boxes show quartiles, with whiskers extending to points within 1.5 times the interquartile range. Boxes are clustered by the (categorical) value of $r_{inh}$ and color-coded by $p_{inh}$. Bottom: Fraction of networks showing significant Pearson’s correlation for the $r_{inh}$-$p_{inh}$ parameter space. Significance was assessed with a two-tailed t-test and Bonferroni correction at *p* < 0.05.

**Fig 8:**
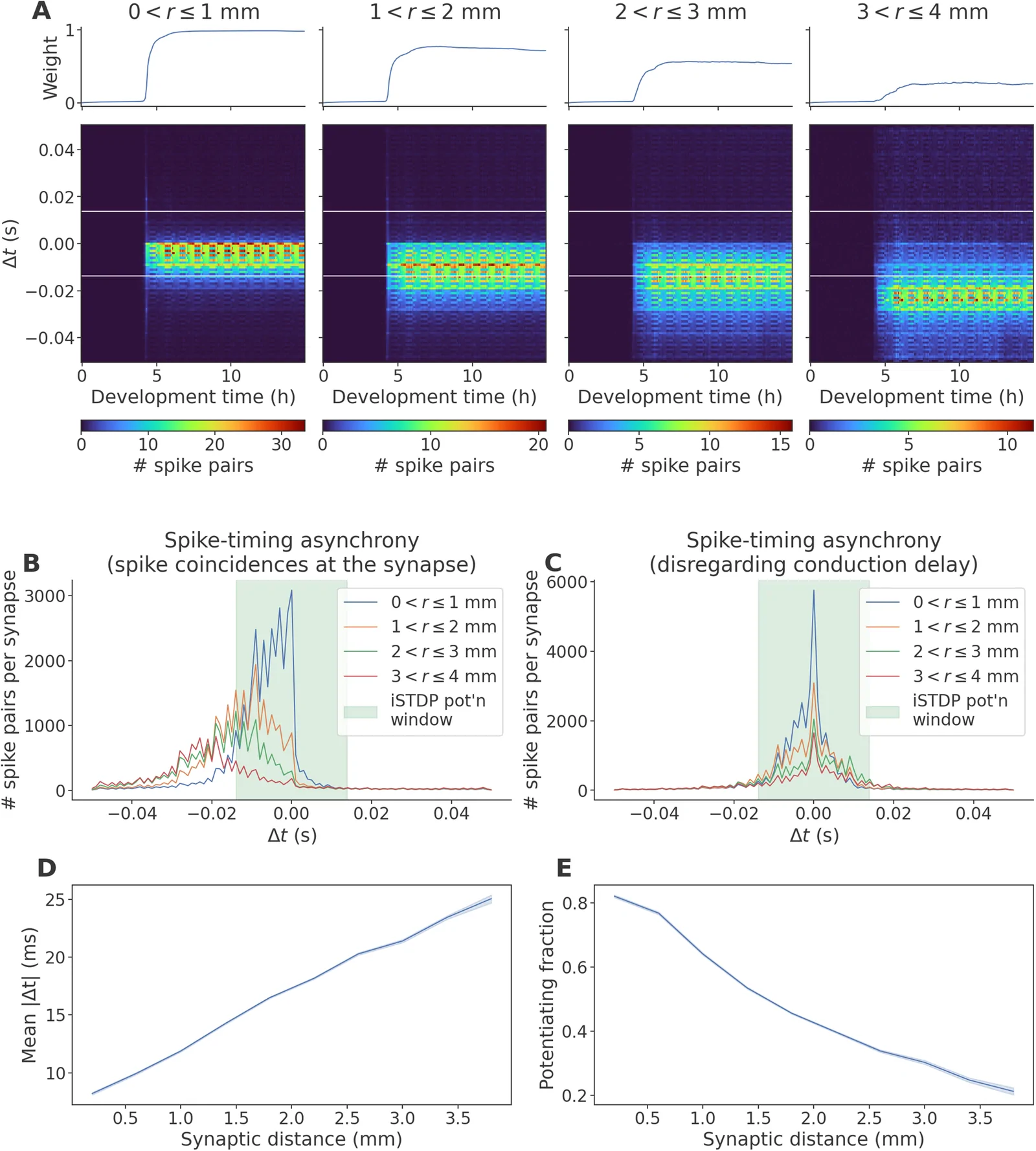
Long-range synapses experience delayed spike synchrony. **(A)** Spike coincidence plots for inhibitory synapses in a sample network ($r_{inh}=\text{4}$ mm, $p_{inh}=\text{0}.\text{7}$), sorted by connection length from the shortest connections (left) to the longest connections (right). The top panel shows the average weight of the connections over development, while the bottom panel shows the density of spikes paired within ±50 ms at their respective $\Delta t=t_{post}-t_{pre}$. The white horizontal lines indicate the boundaries of the potentiation window of the inhibitory STDP rule. Spike timing is measured locally at the synapse, mirroring the STDP weight update procedure. **(B)** The same data summed over development time and plotted as lines. The shaded area indicates the potentiation window of the inhibitory STDP rule. **(C)** The same data but using the global timing of spikes instead of the timing local to each synapse. **(D, E)** Relating synaptic distance, grouped into bins of width 0.4 mm, to spike coincidence across all networks in the final hour of simulation. **D**: Mean absolute spike-timing difference (|Δt|) in each bin. **E**: Fraction of spike pairs within the potentiation window in each bin. Lines show the mean across networks; shaded regions indicate 95% confidence intervals.

### Excitation-inhibition balance in the developed state

That higher inhibitory density leads to lower Δp, as shown above, is not prima facie surprising: Previous work [20] has shown that criticality typically emerges when excitation and inhibition hold each other in balance. This is hinted at in the results presented above, where we have used the initial constraint. To verify the role of E/I balance in maintaining criticality in our model, we analyzed the steady state of the networks following development. After development, most synapses of all types had weight values approaching either 0 or 1 (S3 Fig, the fraction of weights within 0.1≤W≤0.9 is less than 5% in almost all networks). Therefore, we set a weight threshold of 0.5, considering any synapses at or above the threshold as active, and any below as inactive. Then, we counted the number of active excitatory and inhibitory synapses onto each neuron (the effective in-degree $k^{eff}$) and averaged across neurons. Plotting the inhibitory against the excitatory effective degree of all 1250 networks (25 parameter settings, 10 networks, 5 runs), we see a clear linear separation between supercritical networks, whose inhibitory effective degree is low, and subcritical networks, whose inhibitory effective degree is high relative to their excitatory effective degree (Fig 5A). The same is true when considering the sum of excitatory and inhibitory weights per neuron rather than the effective degree (Fig 5B). Extracting just the critical networks (|Δp|<0.1, Fig 5C), we find that a linear relationship fits the data well, with $W_{inh}=4.10W_{exc}-12.76$ and a Pearson correlation coefficient of 0.288 (p = 0.0122, n = 179 critical networks). This linear relationship holds across a range of |Δp| cutoff values (S4 Fig). Finally, to describe the relationship between the E/I balance and the criticality indicator Δp across all networks, we regressed Δp against the logarithm of the weight ratio $W_{exc}itbf/W_{inh}$ (Fig 5D), yielding a significant positive relationship (r = 0.679, p = 4.8e-170, n = 1250). This indicates that, as expected, larger E/I ratios are seen in networks with more supercritical outcomes, and confirms that our model is well-behaved with regards to the emergence (or otherwise) of criticality.

### Effect of spatial constraint

Unlike most previous research, our approach to manipulating networks not only affects connection density or weight, but also network topology based on the spatial relationships between neurons. That is, we wanted to know how the length of inhibitory connections affects criticality independently of connection density. However, the parameters we chose to manipulate the networks, $r_{inh}$ and $p_{inh}$, both affect connection density. Therefore, to cleanly separate the effects of connection length from those of connection density, we calculated the average inhibitory in-degree and the average inhibitory connection length for each network, sliced the resulting space into bins of roughly equal inhibitory degree, and analyzed the effect of connection length separately in each slice as detailed below. Note that, unlike in the above analysis on E/I balance, here we used the connection blueprint, i.e., all existing connections regardless of their weight after development, to quantify the structurally defined connectivity and thereby capture the effect of the initial constraint.

### Long-range inhibitory networks yield larger avalanches

First, we mapped all networks into the space of mean inhibitory degree $k_{inh}$ (Fig 6A) and mean inhibitory connection length $l_{inh}$ (Fig 6B). There is clear clustering of networks, an artifact of the discrete set of parameter values. Plotting $l_{inh}$ against $k_{inh}$ (Fig 6C), we also see an obvious absence of any networks in the lower right half of the plot (high degree with short average length) due to the spatial nature of our model. Considering the dynamics, we see that networks with greater $k_{inh}$ clearly show lower Δp (Pearson’s r = -0.62, p = 6e-136, n = 1250; Fig 6A), corroborating the above findings on E/I balance even when ineffective connections are included.

The relationship between Δp and $l_{inh}$ is similar, but weaker (r = -0.4, p = 2e-48, n = 1250). Noting the dependence between degree and connection length (Fig 6C; networks with high degree necessarily have many long-range connections), we sought to isolate the influence of connection length $l_{inh}$. To do so, we sliced the data into overlapping bins of width 10 along the $k_{inh}$ axis with bin centers at $k_{inh}=\text{5},\text{1}\text{0},\text{1}\text{5},\ldots ,\text{9}\text{0},\text{9}\text{5}$ (see S5 Fig), such that each slice contained networks of approximately equal degree but potentially very different connection lengths. We then correlated $l_{inh}$ in these slices with Δp (see an example in Fig 6D). Surprisingly, and contrary to the relationship in the whole data (Fig 6B), we see that greater $l_{inh}$ in fact predicts greater Δp across all slices that include networks from multiple r/p settings (Figs 6E and S5; fitted slopes are greater than 0 as assessed by a one-sided t-test, t = 4.66, p = 2e-4, n = 13). In other words, networks with longer inhibitory connections are more supercritical than networks with a similar number of shorter inhibitory connections.

We note that the slice-wise positive correlations do not conflict with the overall negative correlation (Fig 6B), but rather show that the overall correlation result is largely due to the confound between $l_{inh}$ and $k_{inh}$. In addition, as we show in Figs 6F and S6, the correlation between $l_{inh}$ and Δp in slices is not mediated by variations in $k_{inh}$ within the slices, which are not related to $l_{inh}$ in a systematic manner (fitted slopes are not significantly different from zero as assessed by a two-sided t-test, t = 1.29, p = 0.22, n = 13).

### Long-range inhibitory connections are less functional

Next, we focused on possible reasons for this relationship. The mediating factor between initial connectivity and critical dynamics after development is, of course, synaptic weight. We analyzed the relationship between average inhibitory (Fig 7A) and excitatory (Fig 7B) weights and the mean length of inhibitory connections $l_{inh}$, controlling for degree as before. We find that longer connections, i.e., higher $l_{inh}$, consistently led to lower inhibitory weight averages, while excitatory weights were unaffected. Put differently, networks with longer-range inhibitory connections developed higher E/I ratios, leading to the more supercritical outcomes observed above.

Why do networks with longer inhibitory connections have lower inhibitory weights? To answer this, we turned to an analysis of individual synaptic weights within networks, correlating inhibitory synaptic weight against the distance between pre- and postsynaptic neurons. As shown in the example in Fig 7C, longer-range inhibitory synapses tend to be weaker than short-range synapses. This holds true across networks in most of the parameter space of $r_{inh}$ and $p_{inh}$ (Fig 7D, top), with an average correlation coefficient of r=−0.32±0.18. The effect is naturally weaker where $r_{inh}$ is so small that there are few inhibitory connections and little variation in their distance to begin with, but very consistent in networks with $r_{inh}\geq \text{2}$ mm (Fig 7D, bottom), where the correlation is very strong on average (r=−0.43±0.18).

A small caveat is warranted here. Due to our choice of a hard-bounds STDP rule, weights developed into a bimodal distribution with values of either nearly 0 or nearly 1 as noted above and shown in S3 Fig (the fraction of weights within 0.1≤W≤0.9 is less than 5% in almost all networks). Consequently, the observed lower inhibitory weight average for networks with more long-range connections (Fig 7A) and the negative correlations between length and weight within networks (Fig 7D) should be interpreted in the sense that longer-range connections are less likely to be potentiated into an active state than short-range ones. Put differently, networks with longer average connection lengths have a smaller pool of functional (short-range) inhibitory synapses, and thus a lower effective inhibitory degree. Together with the finding of relatively tight E/I balance constraints on criticality (Fig 5), this neatly explains the higher values of Δp in longer-range networks (Fig 6C).

### Long-range synapses experience delayed spike synchrony

To understand why long-range connections fail to potentiate over development, we then considered the underlying learning rule, the Mexican hat shaped inhibitory STDP rule, and its driving factor, the coincidence of pre- and postsynaptic spikes. If long-range connections are not being strengthened, it must be the case that there are few coincident spike pairs relative to non-coincident spike pairs. To confirm this and understand how distance affects spike coincidence, we turned to a sample network that reaches criticality ($r_{inh}=\text{4}$ mm, $p_{inh}=\text{0}.\text{7}$, shown also in Fig 7). We separated synapses into groups by their distance, then considered all spike pairs with a temporal separation at the synapse of less than 50 ms, plotting their occurrence over developmental time as a heatmap (Fig 8A).

In all synapse groups, the early developmental period was marked by very low levels of coincident activity, consistent with low overall activity and a lack of growth in inhibitory weights (cf. Fig 3). After the transition to criticality at ~4.5 hours, all groups showed greatly increased coincident spiking, though with a clear trend towards more frequent coincident spikes in shorter-range synapses (note the different color scales in Fig 8A). Crucially, while the timing of pre- and postsynaptic spikes was approximately synchronous in short-range synapses, long-range synapses showed strong asynchrony, with most spike pairings following a post-before-pre pattern (Δt<0). This is shown more clearly still in the histogram over all time in Fig 8B.

To understand this asynchrony, consider that the timing of spike pairs in the above was measured at the synapse, which was modelled at the location of the postsynaptic neuron. Therefore, the presynaptic spike timing included a distance-dependent conduction delay, while the postsynaptic spike was recorded at the time of its emission without delay. Correcting for this delay, i.e., recording as the Δt of a spike pair the time difference of the emission of spikes at the respective neuron rather than that of their arrival at the synapse, we see that all spike pairs, from the shortest-range to the longest-range synapses, strongly tend towards synchrony on average (Figs 8C and S9). Together with the axonal (i.e., presynaptic) delay and the shape of the inhibitory learning rule, this synchrony in spiking therefore leads to potentiation in short-range synapses, but depression in long-range synapses.

To test whether this relationship between synaptic distance and spike-timing asynchrony generalizes across networks and parameter regimes, we quantified the fraction of spike pairs falling within the potentiation window as a function of synaptic distance for all networks, focusing on the final hour of simulation time. For all inhibitory synapses, the mean absolute spike-timing difference |Δt| and the fraction of spike pairs within the potentiation window (relative to all spike pairs separated by less than 50 ms) were computed. Synapses were then grouped into distance bins across all networks. Spike-timing asynchrony increased with distance (Fig 8D), while, as a consequence, the fraction of potentiating spike pairs decreased monotonically with synaptic distance (Fig 8E). A weighted least squares regression of the latter relationship, with weights proportional to the number of synapses per bin and cluster-robust standard errors at the network level, confirmed a strong negative effect of distance (β = −0.219, 95% CI [−0.222, −0.216], p < 10^−16^), while controlling for $p_{inh}$ and $r_{inh}$. These results demonstrate that the reduction in coincident spike timing with distance is a robust, network-level phenomenon.

Consequently, many long-range inhibitory synapses are ineffective with weights close to 0, and therefore, networks with a substantial fraction of long-range inhibitory synapses experience both less inhibition and thus a greater tendency towards supercritical dynamics than networks where those same synapses are concentrated in a smaller radius. Tying this back to our connectivity constraints, we should therefore expect networks with small radius $r_{inh}$ and large connection probability $p_{inh}$ to show fewer large avalanches than corresponding networks with larger $r_{inh}$ and smaller $p_{inh}$. Looking back at Fig 4C, we see exactly this: Networks with $r_{inh}=\text{2}$ mm and $p_{inh}=\text{0}.\text{7}$ (mean $k_{inh}=\text{4}\text{1}$, mean Δp=0.59) are less supercritical than networks with $r_{inh}=\text{3}$ mm and $p_{inh}=\text{0}.\text{5}$ (mean $k_{inh}=\text{4}\text{5}$, mean Δp=0.66), even though the latter have more inhibitory connections.

## Discussion

In this work, we demonstrated that criticality is affected by the distance between inhibitory neurons and their postsynaptic targets, in that long-range inhibitory connections tend to be weaker than short-range connections. Networks with preferentially short-range inhibitory connections are therefore more strongly inhibited, biasing the dynamics towards the subcritical phase. We further showed that synaptic delay is the main cause for the lower weights in long-range synapses, highlighting the role of conduction delays in establishing and maintaining criticality.

While we have explained our findings in terms of effective degree as a result of the strongly bimodal weight distributions observed, related work has employed both hard-bound STDP rules leading to bimodal weight distributions and soft-bound STDP rules leading to more realistic unimodal weight distributions but found no substantial difference in the resulting patterns of activity and criticality [18]. This allows us to speculate that in our case, too, a soft-bound rule would lead to similar outcomes in terms of activity while leading to explanations in terms of graded variations of synaptic weight.

Our model does not demonstrate self-organized criticality, referring to systems that intrinsically evolve towards a critical point, as shown by the narrow band of parameter settings that lead to criticality (if indeed there is any, see below), and consequently the large number of networks with persistently sub- or supercritical dynamics. This stands in contrast to related work, where short-term plasticity [15] or long-term plasticity with high learning rates [18] directly contribute to the establishment and maintenance of criticality in a comparatively broad region of parameter space. With our approach to plasticity, with low learning rates enforcing a scale separation between development and dynamics, we chose to highlight the structural, rather than dynamic, contributions to criticality.

There is a clear connection between our results and cortical connectivity patterns, which are widely known to strongly favor short-range local connections for inhibitory synapses [35]. While this has been attributed in part to a reduction of wiring cost [36,37], our work suggests that short-range connectivity also emerges as a consequence of an interplay between the homeostatic quasi-Hebbian learning rule thought to dominate inhibitory long-term plasticity [29] and the frequent occurrence of neuronal avalanches in near-critical networks. In our model, long-range synapses are not weakened because distant neurons are not acting in synchrony – indeed, on average, they are – but rather because transmission delays hinder the consistent temporal coordination of pre- and postsynaptic events. However, unlike our model, cortex possesses higher-order structure, which may bias activity propagation in ways that allow coordinated firing, and therefore strong inhibitory connectivity, over longer distances along frequently used signaling pathways. More fundamentally, the spatial organization of inhibitory circuits is itself subject to developmental constraints imposed by genetic programs, molecular guidance cues, and activity-dependent maturation processes [38,39]. In this sense, the inhibitory radius in our model may be viewed as an abstract representation of developmental constraints on inhibitory circuit formation.

This interpretation generates a testable hypothesis. If the spatial extent of inhibitory connectivity acts as a developmental constraint on criticality, then genetic or developmental perturbations that alter inhibitory neuron migration, axonal growth, or synaptic targeting should systematically shift network dynamics away from criticality. Specifically, our model predicts that reductions in local inhibitory connectivity, or developmental changes that bias inhibitory connectivity toward longer spatial ranges, should produce more supercritical dynamics due to the reduced efficacy of delayed inhibitory interactions. Conversely, enhanced local inhibitory connectivity should bias networks toward more subcritical dynamics. Such effects may be measurable through changes in avalanche statistics, E/I balance, or related criticality metrics during development. This prediction is particularly relevant to neurodevelopmental disorders such as autism spectrum disorder and schizophrenia, where abnormalities in inhibitory circuit development and E/I balance have been widely reported [40–42].

Related work has shown that higher-order structure in the form of hierarchical modularity is conducive to critical dynamics [43,44]. In some cases, even modularity in only the inhibitory connections is sufficient to promote criticality, while excitatory modularity only plays a secondary role [18]. Relatedly, too many long-range connections of either kind make it difficult for networks to achieve criticality at all [25]. These findings align well with our results, since neural modules or clusters of neurons in close spatial proximity are likely to fire in a coordinated manner, supporting the formation of strong inhibitory synapses. How such modules form in the first place, and what defines their boundaries, however, is outside the scope of our exploration.

A first key limitation of the present model is its relatively small network size. With 100 neurons, our simulations are not suited to establishing scale-free criticality in the thermodynamic sense, and caution is warranted when interpreting avalanche statistics. However, the primary conclusion of this study does not depend on demonstrating asymptotic critical scaling, but rather on identifying systematic shifts in network dynamics across matched configurations. In particular, we show that increasing the spatial range of inhibitory connectivity consistently reduces effective inhibition and biases networks toward more supercritical regimes. This relative comparison is robust across network instances and does not rely on absolute scaling properties. Small-scale spiking networks have been widely used to study mechanisms related to criticality, including models with as few as 32 neurons that nonetheless exhibit signatures of critical dynamics [45], as well as modular network models that emphasize structural determinants of criticality [18]. These studies similarly prioritize mechanistic insight over asymptotic scaling behavior. At the same time, it remains an open question whether the distance-dependent inhibitory effects identified here persist in larger networks or in systems with more complex spatial and modular organization. Future work should therefore test the generality of this mechanism in larger-scale models.

A second key limitation lies in the measures used to gauge criticality in our networks. In the results presented above, we focused on Δp, supplemented with the closely related measure κ, with which we see similar results (S1 Fig). However, both measures rely exclusively on the avalanche size distribution. In recognition of the weakness of these measures, which may easily yield results suggesting criticality where there is, in fact, none, we provide a closer analysis of the critical behavior of our networks in S1 and S9 Figs. Our analysis of Fano factors (Fig 4F and 4G) suggests network states close to criticality throughout the parameter space, but only at very short time scales. In the domain of spike avalanches, we also calculated the scaling exponent relation $\left|\frac{\tau -\text{1}}{\alpha -\text{1}}-\frac{\text{1}}{\sigma \nu z}\right|$ [33,34], a test which many of our networks fail (S1 Fig). Note, however, that this criticality relationship varies with Δpas expected (Fig 4E) and shows a similar trend across the space of $r_{inh}$ and $p_{inh}$, indicating that these measures broadly agree. Further, we note that our results do not depend on true criticality emerging: Rather, the trend towards less supercritical (or more subcritical, or simply more ordered) behavior with greater inhibitory density, but crucially also with longer inhibitory connections, holds in three measures considered (S10 Fig).

Third, we did not evaluate the contribution of short-term depression (STD) to inhibitory connections and criticality. STD has been shown to play a key role in broadening the parameter range for criticality [15,19]. Moreover, STD functions to suppress spurious bursts and activity propagation, thereby preventing supercritical states [26,46]. Although our analysis in Fig 8 suggests that the time window of long-term inhibitory STDP can account for the distance dependence of inhibitory weights, STD may also contribute to this relationship. Understanding how STD and STDP jointly determine criticality will be an important direction for future research.

Whilst we showed that transmission delays have a notable impact on the (effective) structure of a spatially defined network, we have not investigated their dynamical implications. Theoretical considerations have shown that transmission delays affect the time scale of relaxation towards steady state activity when an external input is applied but not the steady state activity itself [24]. In networks of finite size, we suspect that the dynamics may well depend on transmission delays in nontrivial ways and would encourage further research along these lines.

## Supporting information

S1 FigCriticality indicators Δp, κ, and the criticality relation $\left|\frac{\tau -1}{\alpha -1}-\frac{1}{\sigma \nu z}\right|$, calculated in each run of each network from activity between 13 and 15 hours.Top: Box plots in the $r_{inh}$-$p_{inh}$ parameter space. Bottom: Mean (n = 50) in each parameter condition.(TIF)

S2 FigFor two power-law fitting conditions, $S_{max}=T_{max}=5or20$, distribution of Δp across in the $r_{inh}$-$p_{inh}$ parameter space (A, C) and the exponent relation $\left|\frac{\tau -1}{\alpha -1}-\frac{1}{\sigma \nu z}\right|$ plotted against Δp (B, D) are shown.(TIF)

S3 FigFraction of weights in three ranges: W<0.1, 0.1≤W≤0.9, and W>0.9, in the $r_{inh}$-$p_{inh}$ parameter space.Top row: Inhibitory weights. Bottom row: Excitatory weights.(TIF)

S4 FigEffects of the Δp cutoff on the E/I balance analysis of criticality.Left: Dependence of the linear fitting results between $W_{exc}$ and $W_{inh}$ (Fig 5C) on the Δp cutoff. r: correlation between total excitatory weights $W_{exc}$ and total inhibitory weights $W_{inh}$. p: p-value of linear fitting between $W_{exc}$ and $W_{inh}$. Right: Dependence of the linear regression slope on theΔp cutoff.(TIF)

S5 FigScatter plots and associated linear fits relating $l_{inh}$ to Δp in each slice of $k_{inh}$, see Fig 6E.Note that there are no data for the range 75 < *k*_*inh*_ ≤ 85.(TIF)

S6 FigScatter plots and associated linear fits relating $l_{inh}$ to $k_{inh}$ in each slice of $k_{inh}$, see Fig 6F.Note that there are no data for the range 75 < *k*_*inh*_ ≤ 85.(TIF)

S7 FigScatter plots and associated linear fits relating $l_{inh}$ to the inhibitory weight average in each slice of $k_{inh}$, see Fig 7C.Note that there are no data for the range 75 < *k*_*inh*_ ≤ 85.(TIF)

S8 FigScatter plots and associated linear fits relating $l_{inh}$ to the excitatory weight average in each slice of $k_{inh}$, see Fig 7D.Note that there are no data for the range 75 < *k*_*inh*_ ≤ 85.(TIF)

S9 FigSynchronization indicators in inhibitory synapses of various lengths across all networks.Top, fraction of spike pairs occurring within ±5 ms; bottom, deviation of the most frequent pre/post spike time difference from 0 ms. Indicators were calculated within each network separately, then averaged across all 50 networks in each grid cell. Spike timings were recorded from all spike pairs occurring within ±50 ms and calculated without including conduction delays, as in Fig 8C. Grey areas indicate no data (i.e., there were no long-range synapses in short-range parameter settings).(TIF)

S10 FigRegression results relating the structural parameters $p_{inh}$ and $r_{inh}$ to the criticality indicators Δp and κ, the criticality relation $\left|\frac{\tau -1}{\alpha -1}-\frac{1}{\sigma \nu z}\right|$, and the Fano factor scaling exponent $\alpha_{F}$.Regressions were completed one outcome at a time as outcome $x=\beta_{r}r_{inh}+\beta_{p}p_{inh}+\epsilon$. Results from individual networks are plotted as connected point pairs, with summary statistics in the background (box: quartiles, horizontal line: mean). Regression coefficients of Δp, κ, and $\alpha_{F}$ were significantly negative as assessed by one-sided t-tests.(TIF)
