# Supplementary material for: Effects of spatial constraints of inhibitory connectivity on the dynamical development of criticality in spiking networks
Source: PLoS Comput Biol. 2026 Oct 1;22(10):e1014798. doi: 10.1371/journal.pcbi.1014798 (PMC13645236; doi:10.1371/journal.pcbi.1014798)
Supplement: Supplementary file 1 [file pcbi.1014798.s012.pdf]

Reviewer #1

> *1. **Substantially strengthen the justification for the specific inhibitory STDP rule.** The "Mexican hat" rule is central to the findings, yet its selection is not sufficiently motivated. You must provide a more rigorous justification for why this specific, non-standard inhibitory STDP rule was chosen over other proposed models (e.g., purely depressive rules). Discuss how its symmetric, Hebbian nature relates to biological findings and explicitly state why its potentiation window is critical for your delay-based hypothesis, as this is the core mechanism of your proposed effect.*

First, we thank the reviewer for insightful suggestions.

We have added the following description of experimental data reporting symmetric inhibitory STDP:

Line 127 in the Manuscript: “A similar symmetric iSTDP rule has been observed experimentally in the mouse auditory cortex (D’Amour and Froemke, 2015).”

Additionally, a previous study explored how the STDP time window affects criticality in a fully connected network model (Ikeda, N., Akita, D., & Takahashi, H., 2023, *Applied Physics Letters*, 123(2)).

> *2. **Clarify the initial weight initialization protocol and justify the choice of zero weights.** The decision to initialize all synaptic weights to zero is unconventional and requires explicit justification. You must explain how this initial "silent" network state corresponds to a biologically plausible developmental stage (e.g., early postnatal development). Furthermore, detail the exact procedure: are all weights truly zero, or is there a minimal, random initial value to break symmetry? The current description is ambiguous and could significantly impact the initial trajectory of network development.*

We initialized all synaptic weights to zero. Network activity subsequently emerged through noise and STDP mechanisms. We anticipate that these initially absent connections correspond to early developmental stages or silent synapses reported in mammals (Xu, W., Löwel, S., & Schlüter, O. M. (2020). Silent synapse-based mechanisms of critical period plasticity. *Frontiers in cellular neuroscience*, *14*, 213.). We added this motivation in the main text:

Line 171: “to imitate an immature neuronal network or silent synapses (Xu, Löwel, and Schlüter 2020)”

> *3. **Provide a rigorous statistical demonstration that the developed weight distributions are indeed bimodal.** The entire argument regarding "effective degree" rests on the claim that weights are bimodal, clustering near 0 and 1. You must supplement Supplementary Figure 3 with quantitative analyses, such as Hartigan's dip test for unimodality or by showing histograms with clear separation. A simple statement that weights "approach either 0 or 1" is not sufficient evidence for such a foundational claim.*

We have modified Supplementary Figure 3. We now show the fractions of excitatory and inhibitory weights in three ranges: $W < 0.1$, $0.1 \leq W \leq 0.9$, and $W > 0.9$. The fraction of weights within $0.1 \leq W \leq 0.9$ is less than 5% in almost all networks, indicating that the weights are nearly binarized. We have added this description in the main text:

> Line 292: "After development, most synapses of all types had weight values approaching either 0 or 1 (Supplementary Figure 3, the fraction of weights within $0.1 \leq W \leq 0.9$ is less than 5% in almost all networks)."

*4. **Explicitly define and justify the threshold for "criticality" (|Δp| < 0.1).** The choice of this specific threshold is arbitrary and lacks justification. You must provide a statistical or theoretical rationale for this cutoff. Explain how this value was determined and demonstrate its robustness, for instance, by showing that the main conclusions hold across a range of reasonable thresholds.*

We used the criticality threshold only for the E/I balance analysis in Figure 5. We confirmed that the results are robust within a Δp threshold above 0.05 as shown in Revision Figure 1 (Supplementary Figure 4). We have added this description in the main text:

> Line 304: "This linear relationship holds across a range of $|\Delta \boldsymbol{p}|$ cutoff values (Supplementary Figure 4)"

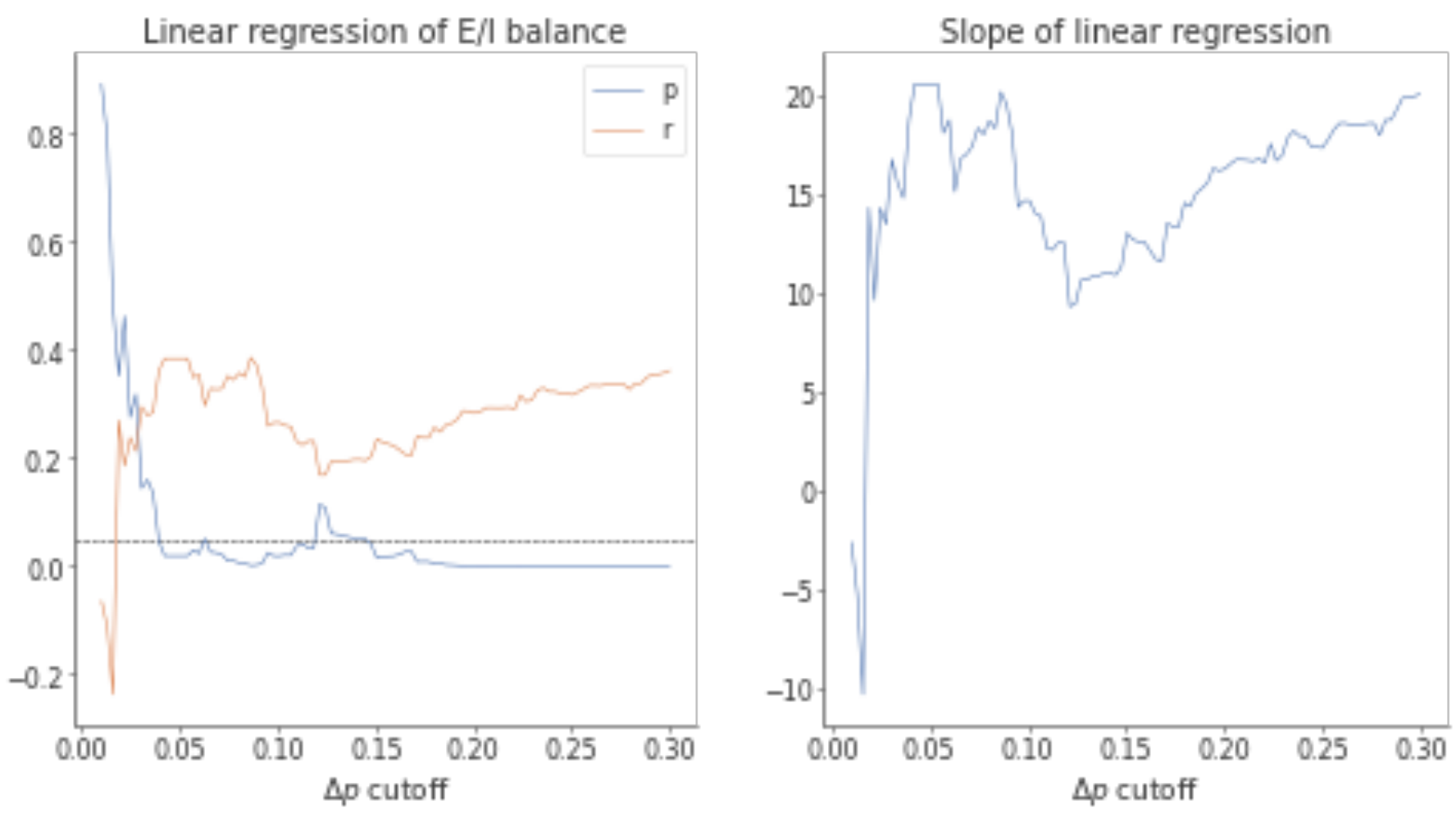

<u>Revision Figure 1:</u> Quantification of how Δp cutoff affects the E/I balance analysis of criticality. r: correlation between total excitatory weights W_{exc} and total inhibitory weights W_{inh}. p: p-value of linear fitting between W_{exc} and W_{inh}.

*5. **Directly address the issue of small network size as a potential confound for criticality analysis.** The 100-neuron network is exceptionally small for reliable criticality analysis. You must explicitly discuss how finite-size effects could influence the observed power laws and the Δp metric. Perform and present a finite-size scaling analysis, or at the very least, provide a robust discussion of how your*

conclusions might be limited by network size and why this model was necessary despite this limitation.

We thank the reviewer for raising this important point. We agree that the relatively small network size limits our ability to make definitive claims about scale-free criticality in the thermodynamic sense. We have revised the Discussion to clarify this limitation explicitly.

Importantly, the main objective of our study is not to establish asymptotic critical scaling, but to identify how spatial constraints on inhibitory connectivity systematically shift network dynamics across matched configurations. Our conclusions are therefore based on relative comparisons, showing that increasing the spatial range of inhibitory connections reduces effective inhibition and biases networks toward more supercritical regimes. This effect is consistently observed across network instances and does not rely on absolute scaling properties.

We also note that small-scale spiking network models have been widely used to investigate mechanisms related to criticality. For example, subsampling and criticality in neuronal networks studied networks with as few as 32 neurons and demonstrated signatures of critical dynamics, while neurobiologically realistic determinants of self-organized criticality in networks of spiking neurons examined how structural features influence criticality in modular networks. These studies similarly emphasize mechanistic insights rather than asymptotic scaling behavior.

To address the reviewer's concern, we now explicitly state in the Discussion that future work should test whether the distance-dependent inhibitory mechanism identified here generalizes to larger networks and more complex spatial structures.

> Line 506: "A first key limitation of the present model is its relatively small network size. With 100 neurons, our simulations are not suited to establishing scale-free criticality in the thermodynamic sense, and caution is warranted when interpreting avalanche statistics. However, the primary conclusion of this study does not depend on demonstrating asymptotic critical scaling, but rather on identifying systematic shifts in network dynamics across matched configurations. In particular, we show that increasing the spatial range of inhibitory connectivity consistently reduces effective inhibition and biases networks toward more supercritical regimes. This relative comparison is robust across network instances and does not rely on absolute scaling properties. Small-scale spiking networks have been widely used to study mechanisms related to criticality, including models with as few as 32 neurons that nonetheless exhibit signatures of critical dynamics (Cramer et al. 2020), as well as modular network models that emphasize structural determinants of criticality (Rubinov et al. 2011). These studies similarly prioritize mechanistic insight over asymptotic scaling behavior. At the same time, it remains an open question whether the distance-dependent inhibitory effects identified here persist in larger networks or in systems with more complex spatial and modular organization. Future work should therefore test the generality of this mechanism in larger-scale models."

*6. **Strengthen the evidence for criticality beyond the avalanche size distribution.** The over-reliance on Δp and κ, both derived from the avalanche size distribution, is*

a major weakness. You must demonstrate that the "critical" networks also satisfy other hallmarks of criticality. The scaling relation (|(τ-1)/(α-1) - 1/σνz|) should be shown to be near zero for your critical networks, not just correlated with Δp. Furthermore, the Fano factor analysis should be presented in the main text to show a power-law scaling over a substantial range of time windows, not just at "very short time scales."

Due to the finite network size and simulation length, there are unavoidable errors in the criticality measurements. We have added the median scaling relation in Figure 4E. The curve exhibits a minimum around Δp = 0, indicating consistency between Δp-based estimates and the scaling relation.

We have also added a Fano factor analysis in Figures 4F and 4G.

*7. **Quantify the relationship between delay and spike-timing asynchrony more rigorously.** The evidence in Figure 8, while illustrative, is qualitative. You must provide a quantitative analysis across all networks. For instance, plot the mean |Δt| or the fraction of spike pairs within the potentiation window as a function of synaptic distance. This will statistically solidify the claim that delay is the primary cause of the observed asynchrony.*

We have added statistical analyses of $|\Delta t|$ in Figures 8D and 8E. In Figure 8D, we show that spike-timing asynchrony increases with distance. In contrast, the fraction of potentiating spike pairs decreases monotonically with synaptic distance (Figure 8E). We have added the following explanation of these results:

> Line 415: "To test whether this relationship between synaptic distance and spike-timing asynchrony generalizes across networks and parameter regimes, we quantified the fraction of spike pairs falling within the potentiation window as a function of synaptic distance for all networks, focusing on the final hour of simulation time. For all inhibitory synapses, the mean absolute spike-timing difference $|\Delta t|$ and the fraction of spike pairs within the potentiation window (relative to all spike pairs separated by less than 50 ms) were computed. Synapses were then grouped into distance bins across all networks. Spike-timing asynchrony increased with distance (Figure 8D), while, as a consequence, the fraction of potentiating spike pairs decreased monotonically with synaptic distance (Figure 8E). A weighted least squares regression of the latter relationship, with weights proportional to the number of synapses per bin and cluster-robust standard errors at the network level, confirmed a strong negative effect of distance (β = −0.219, 95% CI [−0.222, −0.216], $p < 10^{-16}$), while controlling for p_{inh} and r_{inh}. These results demonstrate that the reduction in coincident spike timing with distance is a robust, network-level phenomenon."

*8. **Clarify the analysis of "slices" in Figure 6 and ensure it is not circular.** The method of slicing by kinh to isolate the effect of linh needs a more precise description. Specify the exact binning algorithm (e.g., bin centers, overlaps) and the number of data points in each slice. Ensure that the analysis is not circular, as both*

kinh and linh are derived from the same underlying connectivity parameters (r_inh, p_inh). The analysis must convincingly demonstrate that the effect of linh is independent of the residual variance in kinh within each slice.

We have added a detailed description of our analysis as follows:

> Line 337: "To do so, we sliced the data into overlapping bins of width 10 along the k_{inh} axis with bin centers at $k_{inh} = 5, 10, 15, \ldots, 90, 95$ (see Supplementary Figure 3f), such that each slice contained networks of approximately equal degree but potentially very different connection lengths."

In Figure 6F and Supplementary Figure 6, we showed that $k_{inh}$is not systematically related to l_{inh} within slices, indicating that our analysis is not circular.

*9. **Reconcile the statement that the model does not demonstrate "self-organized criticality" with the manuscript's title and narrative.** You explicitly state that the model does not demonstrate self-organized criticality (SOC) due to the narrow parameter range for criticality. This directly contradicts the title and the narrative of the paper, which focuses on "dynamical development." You must either reframe the manuscript's claims to avoid the term SOC or provide a compelling argument for why this still constitutes a form of self-organization, perhaps by focusing on the weight self-organization towards a balance dictated by spatial constraints.*

We agree with the reviewer that our initial framing of the paper as an example of self-organized criticality was misleading. Accordingly, we have removed the term "self-organized" from the Abstract, Keywords, and Introduction. We retain a discussion of self-organized criticality in the Discussion section to clarify how our results differ from previous studies

*10. **Discuss the potential interaction between short-term depression (STD) and the observed phenomena.** The model includes STD, a known regulator of criticality. You must discuss what role, if any, STD plays in your results. Is the observed distance-dependence of inhibitory weights robust to the removal of STD? A minimal analysis or a thorough discussion of its potential confounding effects is necessary to isolate the effect of delays and long-term plasticity.*

We thank the reviewer for pointing out the possible contribution of STD in the distance-dependence of inhibitory weights. We have added the following discussion about the contribution of STD.

> Line 544: "Third, we did not evaluate the contribution of short-term depression (STD) to inhibitory connections and criticality. STD has been shown to play a key role in broadening the parameter range for criticality (Levina, Herrmann, and Geisel 2007; Zeraati, Priesemann, and Levina 2021). Moreover, STD functions to suppress spurious bursts and activity propagation, thereby preventing supercritical states (Tsodyks and Markram 1997; Golomb and Amitai 1997). Although our analysis in Figure 8 suggests that the time window of long-term iSTDP can account for the distance

dependence of inhibitory weights, STD may also contribute to this relationship. Understanding how STD and STDP jointly determine criticality will be an important direction for future research."

*11. **Improve the clarity and interpretability of several key figures.** Figures 4C, 6C, and 7D, which use circular glyphs and complex color-coding, are difficult to interpret. You must redesign these figures for immediate clarity. Consider using standard scatter plots with error bars or panel arrangements that more intuitively represent the data and the statistical distributions. The reader should not have to spend excessive time deciphering the visualization.*

We have revised the color scheme and combinations in Figures 4C, 6C, and 7D.

*12. **Formulate a more precise and testable hypothesis regarding the link to biological cortex in the discussion.** The discussion currently makes a speculative link to biological inhibitory connectivity. Strengthen this by formulating a specific, testable prediction derived from your model. For example, predict that in cortical circuits, the functional weight of inhibitory synapses should decrease with physical distance, even when controlling for other factors, and propose an experimental method (e.g., paired recordings) to test this prediction.*

We have added a discussion of our hypothesis regarding the cell types and connection distances of cortical inhibitory interneurons.

> Line 477: "More fundamentally, the spatial organization of inhibitory circuits is itself subject to developmental constraints imposed by genetic programs, molecular guidance cues, and activity-dependent maturation processes (Wonders and Anderson 2006; Lim et al. 2018). In this sense, the inhibitory radius in our model may be viewed as an abstract representation of developmental constraints on inhibitory circuit formation.
> This interpretation generates a testable hypothesis. If the spatial extent of inhibitory connectivity acts as a developmental constraint on criticality, then genetic or developmental perturbations that alter inhibitory neuron migration, axonal growth, or synaptic targeting should systematically shift network dynamics away from criticality. Specifically, our model predicts that reductions in local inhibitory connectivity, or developmental changes that bias inhibitory connectivity toward longer spatial ranges, should produce more supercritical dynamics due to the reduced efficacy of delayed inhibitory interactions. Conversely, enhanced local inhibitory connectivity should bias networks toward more subcritical dynamics. Such effects may be measurable through changes in avalanche statistics, E/I balance, or related criticality metrics during development. This prediction is particularly relevant to neurodevelopmental disorders such as autism spectrum disorder and schizophrenia, where abnormalities in inhibitory circuit development and E/I balance have been widely reported (Rubenstein and Merzenich 2003; Nelson and Valakh 2015; Sohal and Rubenstein 2019)."

Reviewer #2

In this manuscript, the authors study how constraints on spatial distribution of inhibitory cells in a network of spiking neurons influences its ability to self-organize to criticality. The work is generally well presented and the idea is of interest for the critical brain dynamics community. I have mostly minor comments and one important major revision that could potentially alter interpretation of some results:

We thank the reviewer for encouraging comments on our manuscript.

Major: Criticality measurements
Criticality measurements are questionable (deviation of data size distribution from power law fit, and deviation from -1.5 power law; also the authors evaluated Fano factor, but the results are only very briefly mentioned in the Discussion and presented on Supp Fig. 2).
1) Great caution needs to be exercised when fitting the avalanche size distributions, specially when one is trying to quantify distance from criticality based on them. I would strongly suggest to perform the fit on log space (fit a linear function to the log of frequency vs the log of sizes). Also, maximum likelihood fitting might be more appropriate. Finally, the cutoff for the distributions must be taken into account and removed from the power law fitting. Results could change qualitatively after a more precise method is employed.

We performed the fitting in log–log space. However, linear fitting over the full range of avalanche sizes and durations failed to accurately capture the distribution, due to deviations from a power law in the large-avalanche regime (Revision Figure 2).

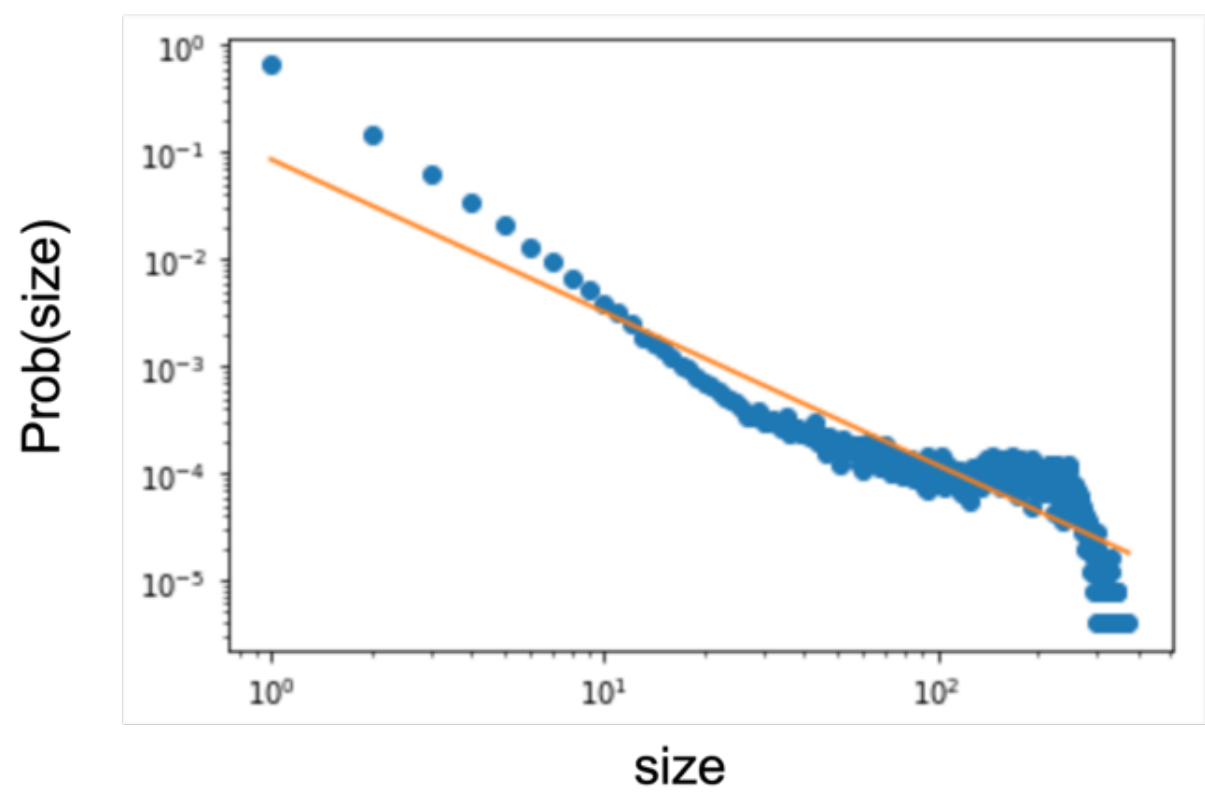

Revision Figure 2: Failed linear fitting in log-space. Blue dots: avalanche size distribution of a super critical network. Orange line: linear fitting in log space.

Therefore, we restricted the fitting range by setting upper bounds of the fitting range ($S_{max} = 10, T_{max} = 10$). We confirmed that varying these maximum size and duration values around 10 qualitatively preserved Δp changes and Δp-γ(DCC) plots, as shown in Supplementary Figure 2. Accordingly, Δp values have been updated in all figures. The results in Figure 4 are qualitatively consistent with those in the

original manuscript. We have added a description of the fitting method in the Method section:

> Line 184 in the Manuscript: "Because the avalanche size distribution is approximately linear in the small-size region, we restricted the fitting range to $S \leq S_{max} = 10$. This approach was adapted from Srinivasan et al. 2024, in which both the small- and large-size tails were excluded from fitting because of their nonlinearity. In our case, only the large-size tail was excluded, as the small-size region remained approximately linear. To assess the robustness of this choice, we also performed sensitivity analyses using $S_{max} = 5, 20$, confirming that the conclusions obtained with $S_{max} = 10$ were not dependent on the specific fitting range selected."

Although the estimated power-law exponents differ from those of the classical branching model, they are comparable to those reported in a recent study of criticality in an excitation–inhibition balanced network model (Yang, Z., Liang, J., & Zhou, C., 2025, *Physical Review Letters*, 134(2), 028401). We have added a discussion about this point:

> Line 536: "Furthermore, the exponent of the avalanche size distribution in our model is approximately 2, which differs from the classical branching process prediction of 1.5. A recent study demonstrated that an excitatory–inhibitory (E/I) balanced neural network without spatial structure can exhibit critical dynamics characterized by a size exponent of around 2 (Z. Yang, Liang, and Zhou 2025). It will be interesting to investigate in future work how our model relates to their framework and whether similar underlying mechanisms account for this deviation from the classical branching model."

2) Finally, the deviation from criticality measure employed (known as DCC in the literature) in Fig. 4E has been shown to give inconsistent results (e.g. Srinivasan et al. 2024), so interpretation must be done carefully here. For example here in Fig. 4E, one could interpret that based on that measure the negative Dp (which the authors interpret as subcritical) is as closer to criticality than the close to zero Dp.

We thank the reviewer for pointing out an important reference about the criticality relation, DCC. After applying the new fitting method, we have added the median scaling relation DCC in Figure 4E. The curve exhibits a minimum around $\Delta p = 0$, indicating consistency between Δp-based estimates and the scaling relation.

Minor:
3) Intro needs work. Some concepts are not explained well (e.g. avalanches on sandpile model are not "triggered in a scale-free manner").

We have revised the text as follows:

> Line 48: "There, avalanche event sizes are scale-free, meaning that …"

4) External drive or "noise" perhaps too high (0.28 Hz intrinsic firing)? What happens if a (order(s) of magnitude) lower value is used?

To the best of our knowledge, there are only a limited number of experimental studies that directly report intrinsic firing rates during early development. Firing rates of approximately 0.01–0.3 Hz have been reported in dissociated mouse cortical cultures (Sun, J. J., Kilb, W., & Luhmann, H. J. (2010). Self-organization of repetitive spike patterns in developing neuronal networks in vitro. *European Journal of Neuroscience*, *32*(8), 1289-1299.) and in mouse medial entorhinal cortex slices (Sheroziya, M. G., von Bohlen und Halbach, O., Unsicker, K., & Egorov, A. V. (2009). Spontaneous bursting activity in the developing entorhinal cortex. The Journal of Neuroscience, 29(39), 12131-12144.), which are of the same order as our noise level, although slightly lower.
From a technical perspective, a lower noise level leads to slower network development and requires longer simulation times. Therefore, we retain this parameter choice in the present study and leave a more detailed exploration for future work.

5) In development examples (Fig. 3), it might be good to add examples of sub, crit and supercritical network development.

We have added the developmental trajectories of three network states (subcritical, near-critical, and supercritical) in Figure 3.

6) Fig. 4: avalanche size distributions need to be log-binned for proper evaluation of cut-offs; this would help distinguish slightly subcritical from critical cases.

We have added the log-binned results in Figure 4B. The shape of the avalanche distribution is consistent with that obtained using linear binning.

7) In Fig. 5B-D, when calculating total weight, is the weight threshold in place? It wasn't clear to me. If so, I would suggest removing it from this analyses, since a great number of weak connections could be as effective in driving a cell as a few strong ones.

The weight threshold was not used for weight totals in Figure 5B-D. We have added this clarification in the caption of Figure 5:

> Line 803: "Total weight of excitatory and inhibitory synapses (without applying the weight threshold), averaged across neurons and plotted against each other in the same manner as in panel A."

8) Include subtitles above Fig. 8B and C to more readily distinguish what is being shown.

We have added subtitles above Figures 8B and 8C.
