# Supplementary material for: Effects of spatial constraints of inhibitory connectivity on the dynamical development of criticality in spiking networks
Source: PLoS Comput Biol. 2026 Oct 1;22(10):e1014798. doi: 10.1371/journal.pcbi.1014798 (PMC13645236; doi:10.1371/journal.pcbi.1014798)
Supplement: Peer review history

# Peer review: Decision Letter 0

**DOI:** 10.1371/journal.pcbi.1014798.r001

**Authors:** Daniele Marinazzo, Prejaas Tewarie

14 Jan 2026

Effects of Spatial Constraints of Inhibitory Connectivity on the Dynamical Development of Criticality in Spiking Networks

PLOS Computational Biology

Dear Dr. Chao,

Thank you for submitting your manuscript to PLOS Computational Biology. After careful consideration, we feel that it has merit but does not fully meet PLOS Computational Biology's publication criteria as it currently stands. Therefore, we invite you to submit a revised version of the manuscript that addresses the points raised during the review process.

Please submit your revised manuscript by Mar 16 2026 11:59PM. If you will need more time than this to complete your revisions, please reply to this message or contact the journal office at ploscompbiol@plos.org. When you're ready to submit your revision, log on to https://www.editorialmanager.com/pcompbiol/ and select the 'Submissions Needing Revision' folder to locate your manuscript file.

Please include the following items when submitting your revised manuscript:

* A letter that responds to each point raised by the editor and reviewer(s). You should upload this letter as a separate file labeled 'Response to Reviewers'. This file does not need to include responses to formatting updates and technical items listed in the 'Journal Requirements' section below.

* A marked-up copy of your manuscript that highlights changes made to the original version. You should upload this as a separate file labeled 'Revised Manuscript with Track Changes'.

* An unmarked version of your revised paper without tracked changes. You should upload this as a separate file labeled 'Manuscript'.

If you would like to make changes to your financial disclosure, competing interests statement, or data availability statement, please make these updates within the submission form at the time of resubmission. Guidelines for resubmitting your figure files are available below the reviewer comments at the end of this letter

We look forward to receiving your revised manuscript.

Kind regards,

Prejaas Tewarie

Academic Editor

PLOS Computational Biology

Daniele Marinazzo

Section Editor

PLOS Computational Biology

**Journal Requirements:**

1) Please ensure that the CRediT author contributions listed for every co-author are completed accurately and in full.

At this stage, the following Authors/Authors require contributions: Felix Benjamin Kern, Takahisa Date, and Zenas C Chao. Please ensure that the full contributions of each author are acknowledged in the "Add/Edit/Remove Authors" section of our submission form.

The list of CRediT author contributions may be found here: https://journals.plos.org/ploscompbiol/s/authorship#loc-author-contributions

2) We ask that a manuscript source file is provided at Revision. Please upload your manuscript file as a .doc, .docx, .rtf or .tex. If you are providing a .tex file, please upload it under the item type u2018LaTeX Source Fileu2019 and leave your .pdf version as the item type u2018Manuscriptu2019.

3) Please provide an Author Summary. This should appear in your manuscript between the Abstract (if applicable) and the Introduction, and should be 150-200 words long. The aim should be to make your findings accessible to a wide audience that includes both scientists and non-scientists. Sample summaries can be found on our website under Submission Guidelines:

https://journals.plos.org/ploscompbiol/s/submission-guidelines#loc-parts-of-a-submission

4) Please upload all main figures as separate Figure files in .tif or .eps format. For more information about how to convert and format your figure files please see our guidelines:

https://journals.plos.org/ploscompbiol/s/figures

5) We have noticed that you have uploaded Supporting Information files, but you have not included a list of legends. Please add a full list of legends for your Supporting Information files after the references list.

**Reviewers' comments:**

Reviewer's Responses to Questions

**Comments to the Authors:**

**Please note here if the review is uploaded as an attachment.**

Reviewer #1: 1. **Substantially strengthen the justification for the specific inhibitory STDP rule.** The "Mexican hat" rule is central to the findings, yet its selection is not sufficiently motivated. You must provide a more rigorous justification for why this specific, non-standard inhibitory STDP rule was chosen over other proposed models (e.g., purely depressive rules). Discuss how its symmetric, Hebbian nature relates to biological findings and explicitly state why its potentiation window is critical for your delay-based hypothesis, as this is the core mechanism of your proposed effect.

2. **Clarify the initial weight initialization protocol and justify the choice of zero weights.** The decision to initialize all synaptic weights to zero is unconventional and requires explicit justification. You must explain how this initial "silent" network state corresponds to a biologically plausible developmental stage (e.g., early postnatal development). Furthermore, detail the exact procedure: are all weights truly zero, or is there a minimal, random initial value to break symmetry? The current description is ambiguous and could significantly impact the initial trajectory of network development.

3. **Provide a rigorous statistical demonstration that the developed weight distributions are indeed bimodal.** The entire argument regarding "effective degree" rests on the claim that weights are bimodal, clustering near 0 and 1. You must supplement Supplementary Figure 3 with quantitative analyses, such as Hartigan's dip test for unimodality or by showing histograms with clear separation. A simple statement that weights "approach either 0 or 1" is not sufficient evidence for such a foundational claim.

4. **Explicitly define and justify the threshold for "criticality" (|Δp| < 0.1).** The choice of this specific threshold is arbitrary and lacks justification. You must provide a statistical or theoretical rationale for this cutoff. Explain how this value was determined and demonstrate its robustness, for instance, by showing that the main conclusions hold across a range of reasonable thresholds.

5. **Directly address the issue of small network size as a potential confound for criticality analysis.** The 100-neuron network is exceptionally small for reliable criticality analysis. You must explicitly discuss how finite-size effects could influence the observed power laws and the Δp metric. Perform and present a finite-size scaling analysis, or at the very least, provide a robust discussion of how your conclusions might be limited by network size and why this model was necessary despite this limitation.

6. **Strengthen the evidence for criticality beyond the avalanche size distribution.** The over-reliance on Δp and κ, both derived from the avalanche size distribution, is a major weakness. You must demonstrate that the "critical" networks also satisfy other hallmarks of criticality. The scaling relation (|(τ-1)/(α-1) - 1/σνz|) should be shown to be near zero for your critical networks, not just correlated with Δp. Furthermore, the Fano factor analysis should be presented in the main text to show a power-law scaling over a substantial range of time windows, not just at "very short time scales."

7. **Quantify the relationship between delay and spike-timing asynchrony more rigorously.** The evidence in Figure 8, while illustrative, is qualitative. You must provide a quantitative analysis across all networks. For instance, plot the mean |Δt| or the fraction of spike pairs within the potentiation window as a function of synaptic distance. This will statistically solidify the claim that delay is the primary cause of the observed asynchrony.

8. **Clarify the analysis of "slices" in Figure 6 and ensure it is not circular.** The method of slicing by kinh to isolate the effect of linh needs a more precise description. Specify the exact binning algorithm (e.g., bin centers, overlaps) and the number of data points in each slice. Ensure that the analysis is not circular, as both kinh and linh are derived from the same underlying connectivity parameters (r_inh, p_inh). The analysis must convincingly demonstrate that the effect of linh is independent of the residual variance in kinh within each slice.

9. **Reconcile the statement that the model does not demonstrate "self-organized criticality" with the manuscript's title and narrative.** You explicitly state that the model does not demonstrate self-organized criticality (SOC) due to the narrow parameter range for criticality. This directly contradicts the title and the narrative of the paper, which focuses on "dynamical development." You must either reframe the manuscript's claims to avoid the term SOC or provide a compelling argument for why this still constitutes a form of self-organization, perhaps by focusing on the weight self-organization towards a balance dictated by spatial constraints.

10. **Discuss the potential interaction between short-term depression (STD) and the observed phenomena.** The model includes STD, a known regulator of criticality. You must discuss what role, if any, STD plays in your results. Is the observed distance-dependence of inhibitory weights robust to the removal of STD? A minimal analysis or a thorough discussion of its potential confounding effects is necessary to isolate the effect of delays and long-term plasticity.

11. **Improve the clarity and interpretability of several key figures.** Figures 4C, 6C, and 7D, which use circular glyphs and complex color-coding, are difficult to interpret. You must redesign these figures for immediate clarity. Consider using standard scatter plots with error bars or panel arrangements that more intuitively represent the data and the statistical distributions. The reader should not have to spend excessive time deciphering the visualization.

12. **Formulate a more precise and testable hypothesis regarding the link to biological cortex in the discussion.** The discussion currently makes a speculative link to biological inhibitory connectivity. Strengthen this by formulating a specific, testable prediction derived from your model. For example, predict that in cortical circuits, the functional weight of inhibitory synapses should decrease with physical distance, even when controlling for other factors, and propose an experimental method (e.g., paired recordings) to test this prediction.

Reviewer #2: In this manuscript, the authors study how constraints on spatial distribution of inhibitory cells in a network of spiking neurons influences its ability to self-organize to criticality. The work is generally well presented and the idea is of interest for the critical brain dynamics community. I have mostly minor comments and one important major revision that could potentially alter interpretation of some results:

Major: Criticality measurements

Criticality measurements are questionable (deviation of data size distribution from power law fit, and deviation from -1.5 power law; also the authors evaluated Fano factor, but the results are only very briefly mentioned in the Discussion and presented on Supp Fig. 2).

1) Great caution needs to be exercised when fitting the avalanche size distributions, specially when one is trying to quantify distance from criticality based on them. I would strongly suggest to perform the fit on log space (fit a linear function to the log of frequency vs the log of sizes). Also, maximum likelihood fitting might be more appropriate. Finally, the cutoff for the distributions must be taken into account and removed from the power law fitting. Results could change qualitatively after a more precise method is employed.

2) Finally, the deviation from criticality measure employed (known as DCC in the literature) in Fig. 4E has been shown to give inconsistent results (e.g. Srinivasan et al. 2024), so interpretation must be done carefully here. For example here in Fig. 4E, one could interpret that based on that measure the negative Dp (which the authors interpret as subcritical) is as closer to criticality than the close to zero Dp.

Minor:

3) Intro needs work. Some concepts are not explained well (e.g. avalanches on sandpile model are not "triggered in a scale-free manner").

4) External drive or "noise" perhaps too high (0.28 Hz intrinsic firing)? What happens if a (order(s) of magnitude) lower value is used?

5) In development examples (Fig. 3), it might be good to add examples of sub, crit and supercritical network development.

6) Fig. 4: avalanche size distributions need to be log-binned for proper evaluation of cut-offs; this would help distinguish slightly subcritical from critical cases.

7) In Fig. 5B-D, when calculating total weight, is the weight threshold in place? It wasn't clear to me. If so, I would suggest removing it from this analyses, since a great number of weak connections could be as effective in driving a cell as a few strong ones.

8) Include subtitles above Fig. 8B and C to more readily distinguish what is being shown.

**********

**Have the authors made all data and (if applicable) computational code underlying the findings in their manuscript fully available?**

The PLOS Data policyrequires authors to make all data and code underlying the findings described in their manuscript fully available without restriction, with rare exception (please refer to the Data Availability Statement in the manuscript PDF file). The data and code should be provided as part of the manuscript or its supporting information, or deposited to a public repository. For example, in addition to summary statistics, the data points behind means, medians and variance measures should be available. If there are restrictions on publicly sharing data or code —e.g. participant privacy or use of data from a third party—those must be specified.

Reviewer #1: None

Reviewer #2: None

**********

PLOS authors have the option to publish the peer review history of their article (what does this mean?). If published, this will include your full peer review and any attached files.

If you choose “no”, your identity will remain anonymous but your review may still be made public.

**Do you want your identity to be public for this peer review?** For information about this choice, including consent withdrawal, please see our Privacy Policy.

Reviewer #1: **Yes:**  Mohammad Hossein Alizadeh Roknabadi

Reviewer #2: **Yes:**  Tiago Lins Ribeiro

[NOTE: If reviewer comments were submitted as an attachment file, they will be attached to this email and accessible via the submission site. Please log into your account, locate the manuscript record, and check for the action link "View Attachments". If this link does not appear, there are no attachment files.]

**Figure resubmission:**

**Reproducibility:**

?>

# Author comment: Author response to Decision Letter 1

**DOI:** 10.1371/journal.pcbi.1014798.r002

30 Jun 2026

Attachment Submitted filename: revision_letter.pdf

# Peer review: Decision Letter 1

**DOI:** 10.1371/journal.pcbi.1014798.r003

**Authors:** Daniele Marinazzo, Prejaas Tewarie

3 Sep 2026

Dear Dr. Chao,

We are pleased to inform you that your manuscript 'Effects of Spatial Constraints of Inhibitory Connectivity on the Dynamical Development of Criticality in Spiking Networks' has been provisionally accepted for publication in PLOS Computational Biology.

Before your manuscript can be formally accepted you will need to complete some formatting changes, which you will receive in a follow up email. A member of our team will be in touch with a set of requests.

Please note that your manuscript will not be scheduled for publication until you have made the required changes, so a swift response is appreciated.

IMPORTANT: The editorial review process is now complete. PLOS will only permit corrections to spelling, formatting or significant scientific errors from this point onwards. Requests for major changes, or any which affect the scientific understanding of your work, will cause delays to the publication date of your manuscript.

Should you, your institution's press office or the journal office choose to press release your paper, you will automatically be opted out of early publication. We ask that you notify us now if you or your institution is planning to press release the article. All press must be co-ordinated with PLOS.

Thank you again for supporting Open Access publishing; we are looking forward to publishing your work in PLOS Computational Biology.

Best regards,

Prejaas Tewarie

Academic Editor

PLOS Computational Biology

Daniele Marinazzo

Section Editor

PLOS Computational Biology

***********************************************************

Reviewer's Responses to Questions

**Comments to the Authors:**

**Please note here if the review is uploaded as an attachment.**

Reviewer #1: accepted

Reviewer #2: Thanks for thoroughly addressing all my remarks. I do have one final comment that I strongly suggest to address before publication. In your revision, you included a comment in the Discussion on the exponent obtained from the size distribution analysis (~2, instead of the expected 1.5 from branching theory). First, I'd like to emphasize that the 1.5 value in the context of a directed percolation universality class is only expected in high-dimensional systems (d = 4+), such as a random network (Munoz et al., PRE 1999). Your simulation is of a 2D network (directed percolation exponent of ~1.3), although given its small size compared to connection range, it is hard to predict where it would fall exponent-wise. The paper you compare your results to (Yang et al., PRL 2025) employs a random network. Furthermore, and more importantly, avalanche size exponents are *known* to vary with binning (a serious limitation of the method, hence my scrutiny in the initial revision with the methods chosen for criticality evaluation - see e.g., Capek et al., Nat Commun 2023), rendering the discussion of possible deviations from expected exponents fruitless. I therefore strongly suggest to either remove the exponent remark or at the very least include this caveat on the publication.

**********

**Have the authors made all data and (if applicable) computational code underlying the findings in their manuscript fully available?**

The PLOS Data policyrequires authors to make all data and code underlying the findings described in their manuscript fully available without restriction, with rare exception (please refer to the Data Availability Statement in the manuscript PDF file). The data and code should be provided as part of the manuscript or its supporting information, or deposited to a public repository. For example, in addition to summary statistics, the data points behind means, medians and variance measures should be available. If there are restrictions on publicly sharing data or code —e.g. participant privacy or use of data from a third party—those must be specified.

Reviewer #1: None

Reviewer #2: None

**********

PLOS authors have the option to publish the peer review history of their article (what does this mean?). If published, this will include your full peer review and any attached files.

If you choose “no”, your identity will remain anonymous but your review may still be made public.

**Do you want your identity to be public for this peer review?** For information about this choice, including consent withdrawal, please see our Privacy Policy.

Reviewer #1: **Yes:**  Mohammad Hossein Alizadeh Roknabadi

Reviewer #2: **Yes:**  Tiago Lins Ribeiro

# Editor report: Acceptance letter

**DOI:** 10.1371/journal.pcbi.1014798.r004

**Authors:** Daniele Marinazzo, Prejaas Tewarie

PCOMPBIOL-D-25-02270R1

Effects of Spatial Constraints of Inhibitory Connectivity on the Dynamical Development of Criticality in Spiking Networks

Dear Dr Chao,

I am pleased to inform you that your manuscript has been formally accepted for publication in PLOS Computational Biology. Your manuscript is now with our production department and you will be notified of the publication date in due course.

The corresponding author will soon be receiving a typeset proof for review, to ensure errors have not been introduced during production. Please review the PDF proof of your manuscript carefully, as this is the last chance to correct any errors. Please note that major changes, or those which affect the scientific understanding of the work, will likely cause delays to the publication date of your manuscript.

Soon after your final files are uploaded, unless you have opted out, the early version of your manuscript will be published online. The date of the early version will be your article's publication date. The final article will be published to the same URL, and all versions of the paper will be accessible to readers.

For Research, Software, and Methods articles, you will receive an invoice from PLOS for your publication fee after your manuscript has reached the completed accept phase. If you receive an email requesting payment before acceptance or for any other service, this may be a phishing scheme. Learn how to identify phishing emails and protect your accounts at https://explore.plos.org/phishing.

Thank you again for supporting PLOS Computational Biology and open-access publishing. We are looking forward to publishing your work!

With kind regards,

Janani Seenivasan

PLOS Computational Biology | Carlyle House, Carlyle Road, Cambridge CB4 3DN | United Kingdom ploscompbiol@plos.org | Phone +44 (0) 1223-442824 | ploscompbiol.org | @PLOSCompBiol
